# Total Ortholog Median Matrix as an alternative unsupervised approach for phylogenomics based on evolutionary distance between protein coding genes

**DOI:** 10.1038/s41598-021-81926-w

**Published:** 2021-02-15

**Authors:** Sandra Regina Maruyama, Luana Aparecida Rogerio, Patricia Domingues Freitas, Marta Maria Geraldes Teixeira, José Marcos Chaves Ribeiro

**Affiliations:** 1 Department of Genetics and Evolution, Center for Biological Sciences and Health, Federal University of São Carlos (UFSCar), São Carlos, SP 13565-905 Brazil; 2 Department of Parasitology, ICB, University of São Paulo (USP), São Paulo, SP Brazil; 3 Vector Biology Section, Laboratory of Malaria and Vector Research, National Institute of Allergy and Infectious Diseases, National Institutes of Health, 12735 Twinbrook Parkway rm 2E32, Rockville, MD 20852 USA

**Keywords:** Evolutionary biology, Molecular evolution, Phylogeny

## Abstract

The increasing number of available genomic data allowed the development of phylogenomic analytical tools. Current methods compile information from single gene phylogenies, whether based on topologies or multiple sequence alignments. Generally, phylogenomic analyses elect gene families or genomic regions to construct phylogenomic trees. Here, we presented an alternative approach for Phylogenomics, named TOMM (Total Ortholog Median Matrix), to construct a representative phylogram composed by amino acid distance measures of all pairwise ortholog protein sequence pairs from desired species inside a group of organisms. The procedure is divided two main steps, (1) ortholog detection and (2) creation of a matrix with the median amino acid distance measures of all pairwise orthologous sequences. We tested this approach within three different group of organisms: Kinetoplastida protozoa, hematophagous Diptera vectors and Primates. Our approach was robust and efficacious to reconstruct the phylogenetic relationships for the three groups. Moreover, novel branch topologies could be achieved, providing insights about some phylogenetic relationships between some taxa.

## Introduction

Reconstruction of phylogenetic relationships has extensively been performed by molecular systematics; in which traditionally, different methods encompassing multiple sequence alignments and tree reconstruction algorithms analyze ribosomal sequences or conserved protein-coding genes^1^. Molecular phylogenetic trees are based on mutations differentially accumulated in orthologous gene pairs, being constructed either with DNA or amino acid sequences. Evolutionary changes in amino acid sequences are useful for long-term evolution information; because they are more conserved than DNA ones as they reflect the selection effects of non-synonymous nucleotide changes on codons^2^. However, choosing the right orthologous pair is not straightforward. Sequences that are very constrained are also very conserved, so no differences between species may be found. On the other hand, sequences that are very divergent can lead to distorted phylogenies.

The post-genomic era has provided large and unprecedent sequence datasets for thousands of organisms across several taxa of the Tree of Life. Consequently, molecular phylogenetics has benefited; phylogenomics has emerged as a relevant field, integrating molecular evolutionary analyses with genomic data^3,4^. Methods such as supertree^5,6^, supermatrices^7–9^, mega-phylogeny^10^ and multispecies coalescent model^11,12^ have been applied to reconstruct large phylogenies in a way that multi-gene phylogenies represent collectively a single evolutionary landscape.

Each method mentioned differs in some or many points among them, but all of them share the principle of combining individual gene phylogenies to plot a representative phylogenetic tree. Briefly, the supertree method relies on the compilation of topologies from several source gene trees for producing a single tree, whereas the supermatrix method is based on building a large multiple sequence alignment for simultaneous analyses of a giant phylogenetic matrix. Mega-phylogeny method is derivative from the latter, with some improvements during construction of multiple sequence alignments. Lastly, coalescent-based species tree method integrates population genetics processes with mathematical model to deal with heterogeneity and incongruity of gene trees to build a single tree.

Here, we present TOMM (Total Ortholog Median Matrix) as an alternative approach for phylogenomics, in which we propose the use of all orthologous pairs from the desired species for building a matrix based on their median amino acid distance obtained from the proteome (i.e., protein sequences of all protein-coding genes from a genome). Thus, we obtain a phylogeny based on the orthologous forest of sequences (an unsupervised strategy) rather than sets of trees knowingly selected (a supervised strategy).

TOMM retrieves orthologous proteins by using the Reciprocal Smallest Distance (RSD) method, which provides evolutionary distance measures used to build a distance matrix to obtain comprehensive phylograms. To evaluate the efficiency of such new approach, we have tested TOMM in three eukaryote groups of organisms: Kinetoplastida protist, Diptera hematophagous insects, and human and non-human Primates. We used these emblematic groups because of their relevance in the association among the taxa related to parasite-vector-host interaction. Moreover, this triad covers, in a modest way, a reasonable and feasible diversity of eukaryotes, including unicellular, invertebrate, and higher vertebrate organisms.

Kinetoplastid protists are flagellate excavates belonging to the phylum Euglenozoa. The members of the Kinetoplastea are characterized by the presence of circular DNA network disks (called kDNA) inside a large mitochondrion. This group presents a great biological variety, from free-living to parasitic organisms. Most known members belong to the family Trypanosomatidae, which are all obligate endoparasitic, comprising either monoxenous (single host, restricted to invertebrates) or dixenous (two hosts, a vertebrate or plant and an invertebrate vector) life cycles. The family Trypanosomatidae comprises 22 genera distributed in six formally recognized subfamilies^13^. Although most trypanosomatid genera are monoxenous, being able to infect only insects, this family is well known because of the dixenous genera *Leishmania* and *Trypanosoma*, which comprise species pathogenic to humans, causing serious insect-borne infectious diseases, such as leishmaniasis and Chagas’s disease, respectively. Because of the medically important species and their biological diversity, kinetoplastids represent an interesting model for understanding the evolution of both parasitism and pathogenicity.

The blood feeding habit evolved independently multiple times among the 400 hematophagous arthropod genera (over 14,000 species)^14^, including within the Diptera where it developed independently within the Brachycera (tsetse and tabanid flies), and at least twice in the suborder Nematocera to produce the mosquitoes and sand flies. These organisms are vectors of leishmaniasis, African trypanosomiasis, malaria, filariasis, and several viral diseases such as yellow fever, dengue, and zika.

Closing the triad, we performed the TOMM approach in higher vertebrates, represented herein by the Primates order, which is one of the most diverse among the mammals, comprising over 470 species^15^. Primates present extraordinary variations regarding ecological, behavioral, morphological, and evolutionary aspects. Genomic and genetic characterizations of primates are not only important for species conservation and evolutionary insights^16,17^, but also for understanding human evolution and genome structure from a biomedical perspective [reviewed in^18^]. Indeed, evolutionary genomics of host–pathogen interaction has been considered a trait for molecular phylogeny, and correlations between immunity against infections and Primates evolution have been targeted to understand how viral, bacterial, and parasitic diseases emerged to elucidate their different manifestations depending on host species^19^.

Overall, we implemented the TOMM phylogenomic approach for the three focal groups of organisms. The TOMM resulting trees are in good agreement with latest phylogenetic thoughts for the three groups of organisms.

## Results and discussion

**Figure 1:**
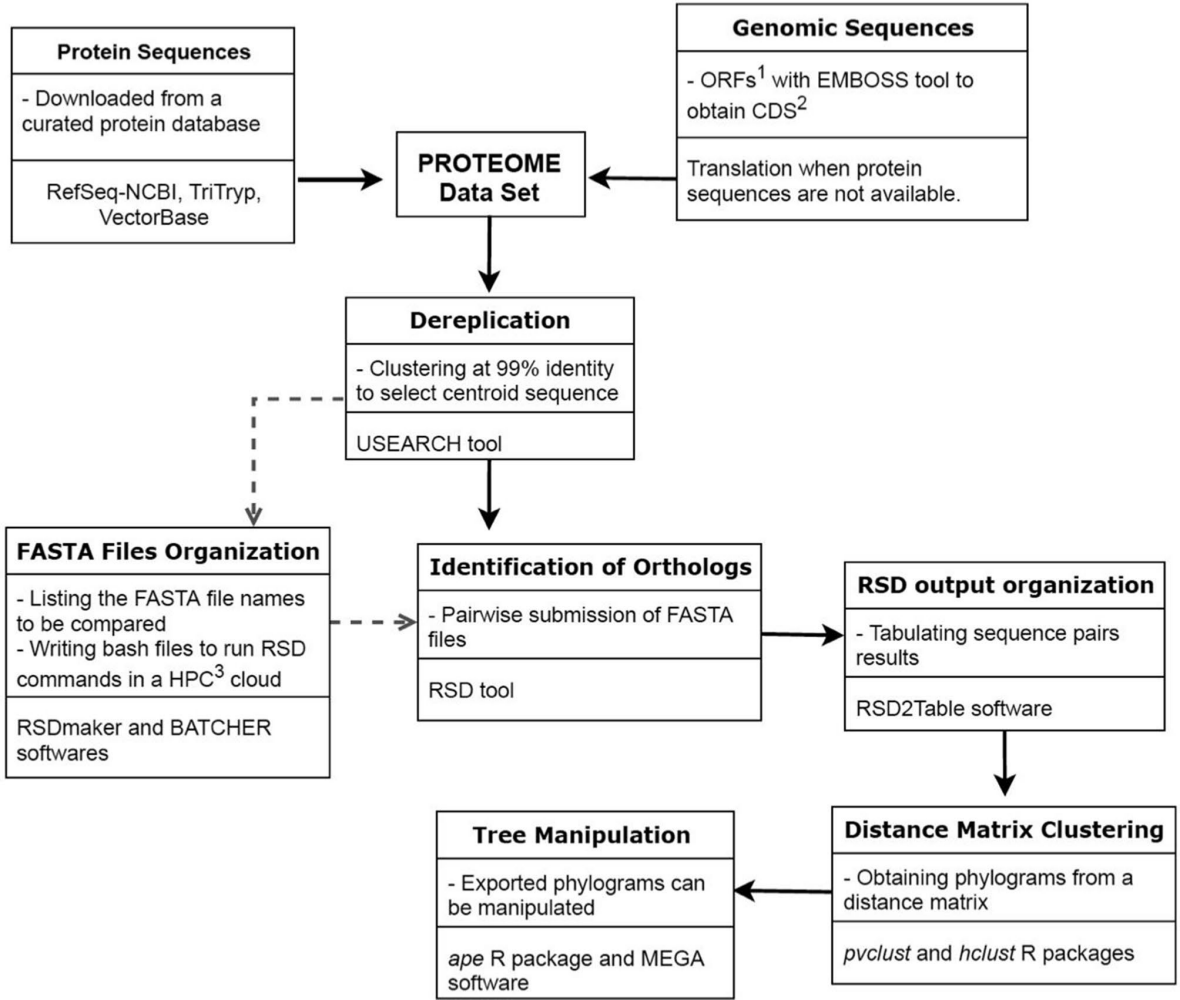
Workflow of TOMM approach for Phylogenomics. Main procedures are depicted, along with used software in each step. 1: open reading frame; 2: coding sequences; 3: High-Performance Computing.

**Figure 2:**
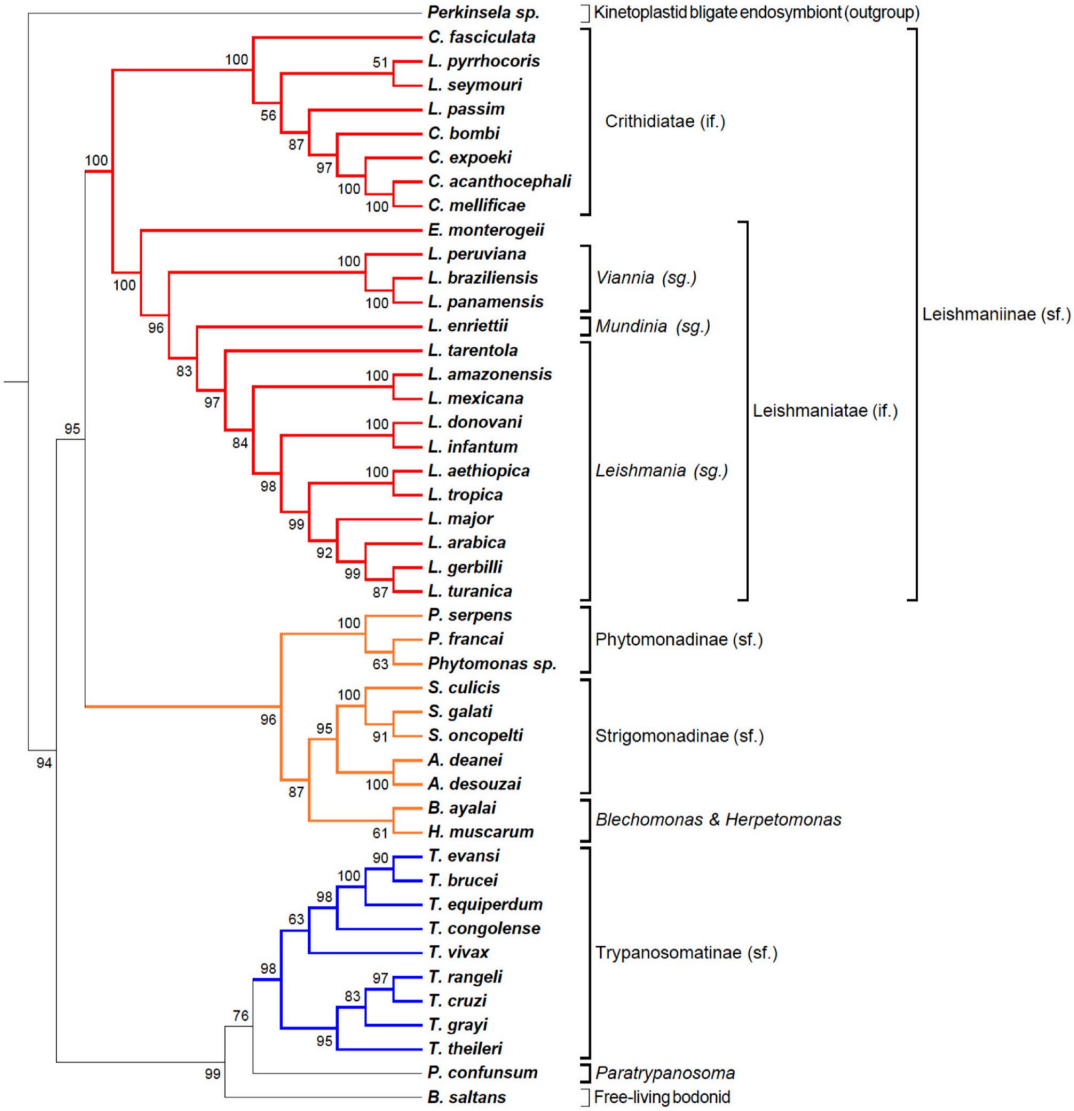
Phylogenomic tree of Kinetoplastid protozoa. Phylogram constructed with the TOMM approach using approximately 5636 orthologous protein pairs across 46 Kinetoplastida species with genome sequence available (Table 1 and Supplemental Table S1). Numbers next to the branches represent the percentages of approximate unbiased support probabilities for 10,000 bootstraps, calculated using the pvclust package^82^ in R (R Core Team. R: A language and environment for statistical computing. R Foundation for Statistical Computing, Vienna, Austria, 2018, URL: https://www.R-project.org/). The Newick file was annotated using the program MEGA 6. Abbreviations: if (infra family); sg (subgenus); sf (subfamily).

**Figure 3:**
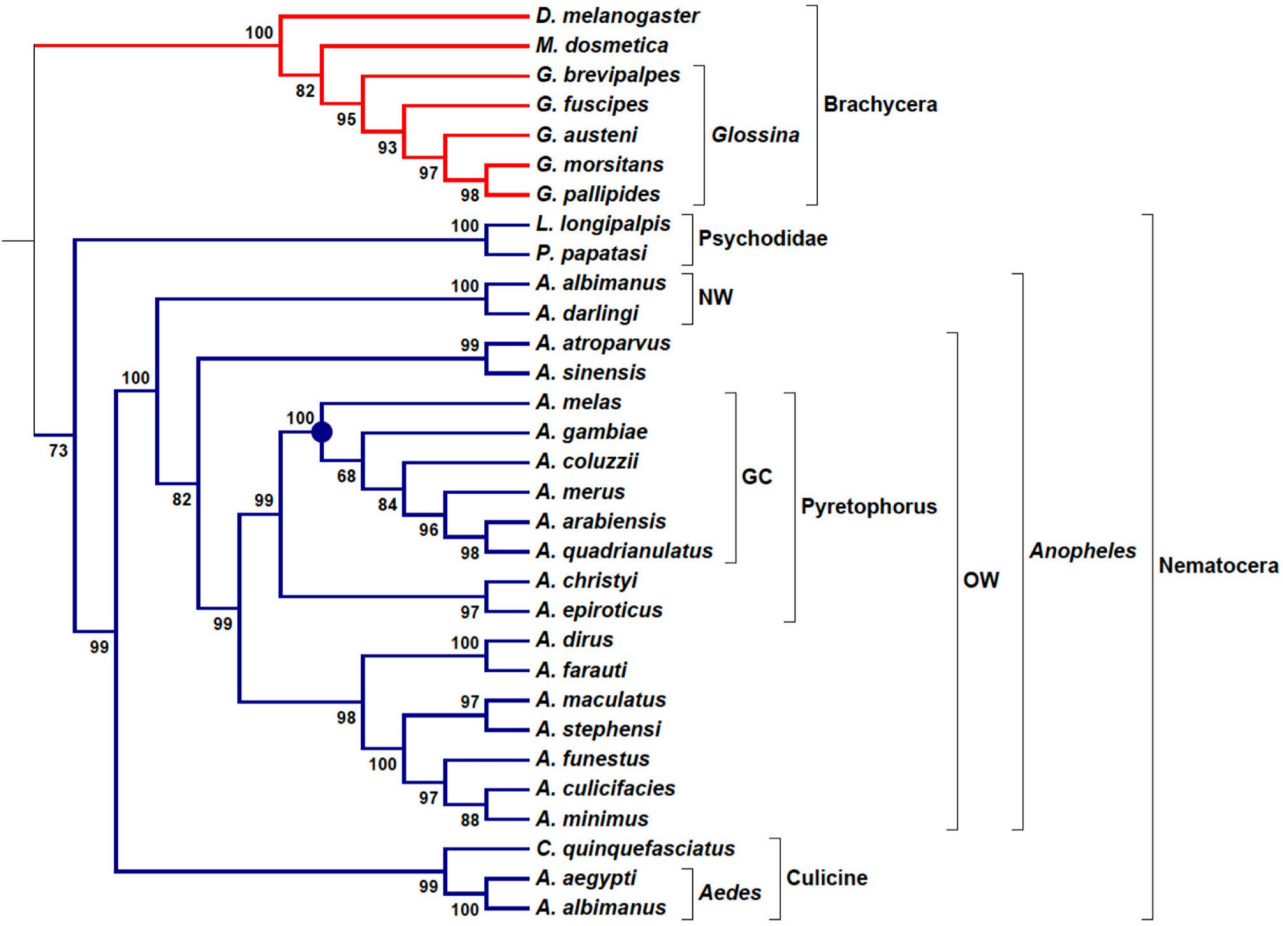
Phylogenomic tree of hematophagous Diptera. Phylogram constructed with the TOMM approach using approximately 8168 orthologous protein pairs across 31 Diptera species with genome sequence available (Table 2 and Supplemental Table S2). Numbers next to the branches represent the percentages of approximate unbiased support probabilities for 10,000 bootstraps, calculated using the pvclust^82^ package in R (R Core Team. R: A language and environment for statistical computing. R Foundation for Statistical Computing, Vienna, Austria, 2018, URL: https://www.R-project.org/). The Newick file was annotated using the program MEGA 6. *NW* New World, OW Old World, *GC* gambiae complex.

**Figure 4:**
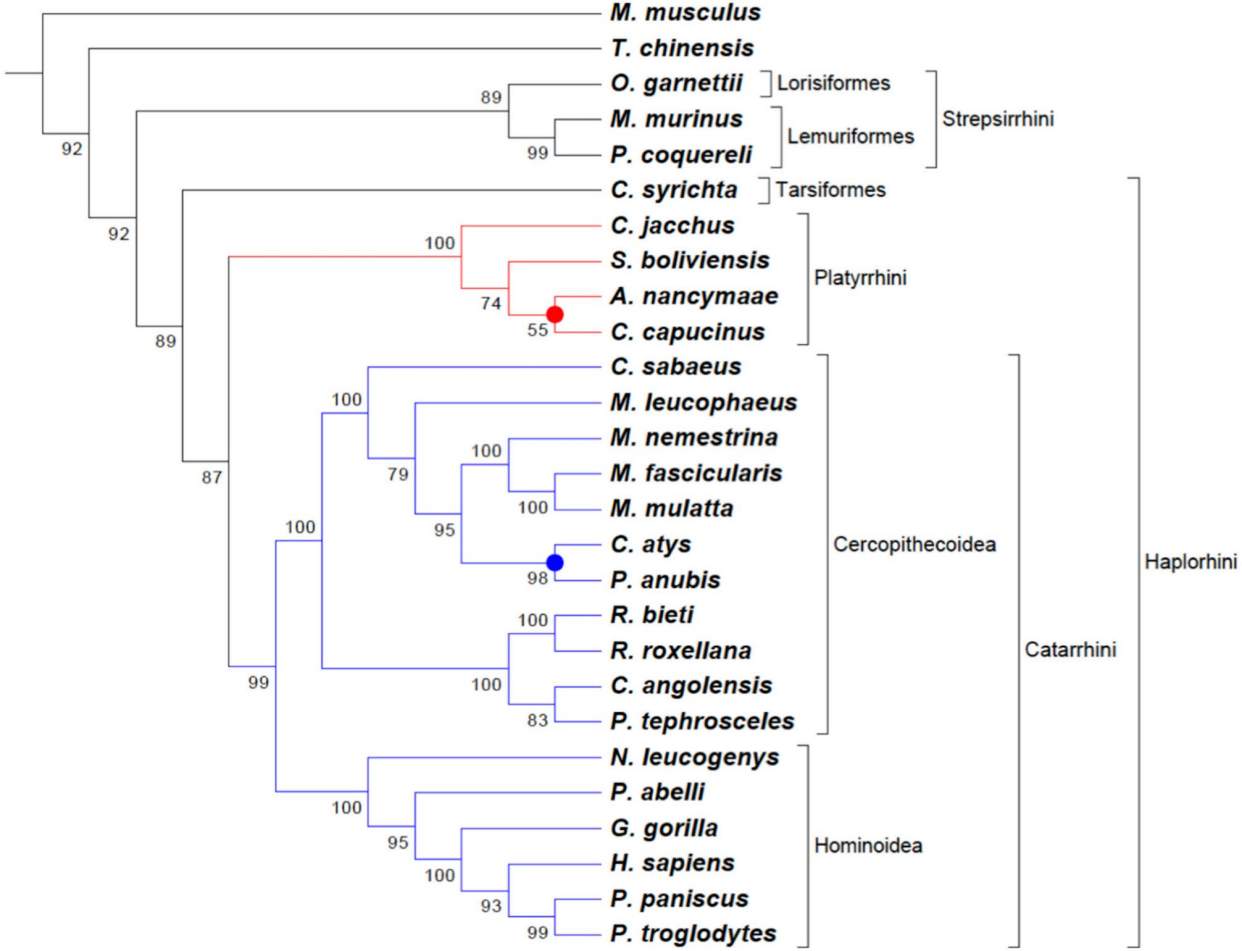
Phylogenomic tree of Primates. Phylogram constructed with the TOMM approach using approximately 23,826 orthologous protein pairs across 25 Primates species with genome sequence available (Table 3 and Supplemental Table S3), and two outgroup species. Numbers next to the branches represent the percentages of approximate unbiased support probabilities for 10,000 bootstraps, calculated using the pvclust^82^ package in R (R Core Team. R: A language and environment for statistical computing. R Foundation for Statistical Computing, Vienna, Austria, 2018, URL: https://www.R-project.org/). The Newick file was annotated using the program MEGA 6.

The overall procedure of TOMM approach is diagramed in Fig. 1. TOMM efficiently recovered known phylogenetic relationships and additionally was able to provide new phylogenetic insights. The three data sets analyzed herein produced well-resolved phylogenies. The Kinetoplastid tree (Fig. 2) showed congruent results with the most recent studies on this group^13,20,21^, with additional new possible relationships between some genera. Similarly, the hematophagous dipteran tree (Fig. 3) resembled the most recent phylogenetic relationships considered for the vectors of Malaria, viral diseases, leishmaniasis, and sleeping sickness^22^. For the Primates, TOMM phylogeny revealed two main clades, separating the most primitive primates (Strepsirrhini) from the other ones (Haplorrhini), that include Tasiiformes and Simiiformes. Among the haplorrhines, Platyrrhini formed a distinct well-supported clade from Catarrhini (Fig. 4), as expected^18,23^. However, TOMM was not efficient in recovering *Cebus* and *Saimiri* as a single clade of Cebidae family, clustering *Cebus* and *Aotus* in a non-supported clade (a.u. 55). Similarly, non-expected results were observed to *C. atys* and *P. nubis*, though with a high probability support (a.u. 98). The resulting trees are described and discussed in more detail hereafter.

### Kinetoplastid tree

In the past decades, molecular phylogenetics using rRNA sequences and protein sequences have shed light in the evolutionary biology of this group, showing that parasitism evolved several times inside Kinetoplastea^13,24^. Comparative genomics of dixenous and monoxenous trypanosomatids were compared to the free-living kinetoplastid, *Bodo saltans*, thought to be the closest relative of the trypanosomatids^13,20^.

Important phylogenomics studies brought up key phylogenies across representative kinetoplastids from genera *Leishmania*, *Trypanosoma*, *Phytomonas, Leptomonas,* and *Bodo*^20^. More recently, such analyses were expanded to over 30 species encompassing several members for each life cycle: free-living, monoxenous, and dixenous parasites^13^. Collectively, these phylogenies were constructed using some gene families and a core of 64 conserved proteins. The TOMM approach has already been applied to Trypanosomatidae family in a study that identified a new parasite found in a fatal case of visceral leishmaniasis, where 36 genomes from trypanosomatids were used^25^. Here, we presented a phylogenomic analysis of kinetoplastids based on at least 1473 orthologous proteins across 45 species with published genomes, comprising flagellates of all lifestyles (see Table 1).

**Table 1:** Characteristics and source of genome-derived proteomes of kinetoplastids used in this work.

Species name	Life cycle	Genome source	Protein sequence source^1^	Publication	Genome size (Mb)	Number of sequences^2^
*Angomonas deanei*	Endosymbiont-bearing monoxenous	NCBI	Orf/blastx REFSEQ	PMID23560078	23	6255
*Angomonas desouzai*	Endosymbiont-bearing monoxenous	NCBI	Orf/blastx REFSEQ	PMID21420905	24.3	6282
*Blechomonas ayalai*	Monoxenous	TriTrypdb	TriTrypdb	PMID27021793	N/A	8037
*Bodo saltans*	Free living	NCBI	NCBI	PMID19068121	39.9	17,840
*Crithidia acanthocephali*	Monoxenous	NCBI	Orf/blastx REFSEQ	PMID23560078	33.8	11,800
*Crithidia bombi*	Monoxenous	NCBI	Orf/blastx REFSEQ	PMID29304093	31.4	7675
*Crithidia expoeki*	Monoxenous	NCBI	Orf/blastx REFSEQ	PMID29304093	34	10,254
*Crithidia mellificae*	Monoxenous	NCBI	Orf/blastx REFSEQ	PMID24743507	58.7	7660
*Crithidia fasciculata*	Monoxenous	TriTrypdb	TriTrypdb	N/A	41.3	9489
*Endotrypanum monterogeii*	Dixenous	TriTrypdb	TriTrypdb	N/A	32.5	8285
*Herpetomonas muscarum*	Monoxenous	NCBI	Orf/blastx REFSEQ	N/A	30.8	10,297
*Leishmania amazonensis*	Dixenous	Unicamp	Unicamp	PMID23857904	31.3	7316
*Leishmania arabica*	Dixenous	TriTrypdb	TriTrypdb	N/A	31.3	8646
*Leishmania aethiopica*	Dixenous	TriTrypdb	TriTrypdb	N/A	32.6	8722
*Leishmania enriettii*	Dixenous	TriTrypdb	TriTrypdb	N/A	30.8	8731
*Leishmania gerbilli*	Dixenous	TriTrypdb	TriTrypdb	N/A	31.4	8599
*Leishmania braziliensis*	Dixenous	NCBI	NCBI	PMID26384787	35.2	8151
*Leishmania donovani*	Dixenous	NCBI	NCBI	PMID22038251	32.4	7960
*Leishmania infantum*	Dixenous	NCBI	NCBI	PMID29273719	32.4	8141
*Leishmania major*	Dixenous	NCBI	NCBI	PMID16020728	32.3	8306
*Leishmania mexicana*	Dixenous	NCBI	NCBI	PMID26452044	32.1	8137
*Leishmania panamensis*	Dixenous	NCBI	NCBI	PMID25707621	31	7742
*Leishmania peruviana*	Dixenous	NCBI	Orf/blastx REFSEQ	PMID26384787	33.4	7155
*Leptomonas pyrrhocoris*	Monoxenous	NCBI	NCBI	PMID27021793	30.4	9284
*Leptomonas seymouri*	Monoxenous	NCBI	NCBI	PMID26317207	27.1	8485
*Leishmania tarentolae*	Dixenous	TriTrypdb	TriTrypdb	N/A	N/A	8305
*Leishmania tropica*	Dixenous	TriTrypdb	TriTrypdb	N/A	32.3	8824
*Leishmania turanica*	Dixenous	TriTrypdb	TriTrypdb	N/A	32.2	8608
*Trypanosoma evansi*	Monoxenous	TriTrypdb	TriTrypdb	N/A	N/A	12,838
*Lotmaria passim*	Monoxenous	NCBI	NCBI	PMID26146231	27.7	4850
*Perkinsela* sp.	Obligate endosymbiont	NCBI	NCBI	PMID28916813	9.5	5192
*Phytomonas francai*	Dixenous (plants)	NCBI	Orf/blastx REFSEQ	PMID28082482	17.7	6410
*Phytomonas* sp.	Dixenous (plants)	NCBI	NCBI	PMID24516393	18	4905
*Phytomonas serpens*	Dixenous (plants)	TriTrypdb	TriTrypdb	N/A	25.7	7329
*Strigomonas galati*	Endosymbiont-bearing monoxenous	NCBI	Orf/blastx REFSEQ	PMID24015778	27.2	6785
*Strigomonas culicis*	Endosymbiont-bearing monoxenous	NCBI	Orf/blastx REFSEQ	PMID23560078	25.4	6778
*Strigomonas oncopelti*	Endosymbiont-bearing monoxenous	NCBI	Orf/blastx REFSEQ	PMID24015778	25	9642
*Trypanosoma brucei*	Dixenous	NCBI	NCBI	PMID16020726	26.5	8132
*Trypanosoma congolense*	Dixenous	NCBI	NCBI	N/A	39.2	19,062
*Trypanosoma cruzi*	Dixenous	NCBI	NCBI	PMID24482508	30.4	7659
*Trypanosoma equiperdum*	Dixenous	NCBI	NCBI	PMID28138343	26.2	10,001
*Trypanosoma grayi*	Dixenous	NCBI	NCBI	PMID25977781	20.9	10,576
*Trypanosoma rangeli*	Dixenous	NCBI	NCBI	PMID25233456	18.1	7331
*Trypanosoma theileri*	Dixenous	TriTrypdb	TriTrypdb	N/A	29.8	11,312
*Trypanosoma vivax*	Dixenous	TriTrypdb	TriTrypdb	N/A	24.7	11,362
*Paratrypanosoma*	Monoxenous	NCBI	NCBI	PMID29078369	27.5	9606

1. Protein sequences were obtained from NCBI, TriTrypDB, or deduced from genome by obtaining open reading frames and adjusting starting Met by blastx to Protozoa-Refseq NCBI database; 2. After clusterization at 99% and removal of sequences smaller than 50 aa. N/A: not available.

The resulting tables of pairwise orthologs were sorted to find the median value of the amino acid distance and thus populate a pairwise matrix (Supplemental Table 1, sheet “AA distance”). The minimum number of ortholog sequences found in the condition above was 1473, found for the *Perkinsela sp.*/*Phytomonas serpens* pair, and the maximum was 8434 sequences, found for the *Crithidia spp./Leptomonas pyrrhocoris* proteome pair (Supplemental Table 1, sheet “Number-50”). This matrix was submitted to the program Pvclust, which provides statistical evaluation of the tree nodes expressed as approximately unbiased (a.u.) α values, where α = 1 − P. The phylogram was exported as a Newick file, including the a.u. values and annotated using the program MEGA 6.

The resulting phylogram built using the total proteome median matrix from kinetoplastid species harboring bacterial endosymbionts, free-living, monoxenous, and dixenous parasites is shown on Fig. 2. The enigmatic non-flagellated kinetoplastid *Perkinsela spp*, an obligate endosymbiont of *Paramoeba* (an amoeba genus considered an opportunistic pathogen of fish and marine invertebrates)^26,27^, is a clear outgroup. Usually, the free-living *Bodo saltans*, the closest known free-living relative of trypanosomatids, served as an outgroup for phylogenomics of trypanosomatids^13,20^. This Bodonidae species was placed in a sister position to *Trypanosoma,* whereas previous phylogenomic studies based on 64 well-conserved proteins strongly supported (1/100 BI posterior probabilities and ML bootstrap values) the late emergence of trypanosomatids as a sister group of *Bodo saltans* (Eubodonida)^28^. Here we were able to provide a higher statistical probability node support (a.u. 99), based on an average of 4999 orthologous proteins with a minimum (1833) and maximum (6022) ortholog pairs with *Perkinsela spp* and *Trypanosoma theileri*, respectively (Supplemental Table 1). In our analysis, between *Trypanosoma* spp. and *B. saltans* (free-living) is placed the monoxenous *Paratrypanosoma confunsum,* an early-branching trypanosomatid. In previous analyses, *P. confusum* branched at the base of the family Trypanosomatidae, representing a link between the ancestral free-living bodonids and the parasitic trypanosomatids^13,21,29^. The heterogeneity composition regarding the kinetoplastid life cycles make this clade a valuable source of information to elucidate the evolution of parasitism.

Corroborating the most recent expanded phylogeny tree of trypanosomatids from Lukes et al. (2018), the phylogram indicates the existence of two subfamilies with strong statistical support, Trypanosomatinae and Leishmaniinae. The Trypanosomatinae includes the parasites from the genus *Trypanosoma*, all dixenous species excepting *T. evansi* and *T. equiperdum*^24^, with important pathogens for humans and livestock. *Trypanosoma vivax* occupies a basal position within a clade with *T. brucei,* representing the African trypanosomes pathogenic to ungulates. The basal position of *T. vivax* in the clade is in accordance with previous results^30,31^.

The Leishmaniinae subfamily comprises two major Sections, Euleishmania (*Leishmania*) and Paraleishmania (*Porcisia and Endotrypanum*)^32–35^. The two sister clades, representing *Euleishmania* and *Paraleishmania,* were recently proposed as the infrafamily Leishmaniatae, whereas Crithidiatae infrafamily comprises all other genera of Leishmaniinae: *Crithidia, Leptomonas, Lotmaria, Novymonas, Zelonia, and Borovskiya*^36,37^.

The “Crithidiatae” clade is composed of monoxenous species and presented maximum statistical node support (a.u. 100), but subclades composed by *Leptomonas, Lotmaria,* and *Crithidia* species resulted from low node support (a.u. 56 and a.u. 51), with *Crithidia fasciculata* clustered apart from the *Crithidia* clade composed of *C. bombi*, *C. expoeki*, *C. acanthocephali,* and *C. mellificae* (a.u. 97). This reinforces the non-monophyletic origins of *Crithidia* members, and the revision of *Crithidia* genus as claimed by others^38,39^. Although monoxenous, Crithidiatae members, such as *Leptomonas,* have been detected in humans as co-infections in visceral leishmaniasis clinical cases^40–42^.

The Leishmaniatae (all dixenous) are clearly divided into the *Leishmania* and *Viannia* sub-genera, with *L. tarentolae*, a lizard parasite, occupying the most basal position in the *Leishmania* subgenus. Previously, this species was classified in the subgenus *Sauroleishmania* but was later shown from molecular phylogenetics to be closer to members of the *Leishmania* subgenus^43,44^. *Leishmania enriettii* from the subgenus *Mundinia* is located between the *Viannia* and *Leishmania* subgenera, as inferred by other phylogenetic studies^45–47^*.* However phylogenetic analyses, including other members of *Mundinia,* such as *L.* (*Mundinia*) *martiniquensis* and *L. (M.) macropodum,* support the most basal position of this subgenus in the genus *Leishmania*^33–35^. *Endotrypanum* is the only known kinetoplastid able to infect erythrocytes of their mammalian host (sloths)^48^. In the present study, *E. monterogeii* clearly clustered apart from all other subgenera of Leishmania as observed previously^33–35^. Between the clades Trypanosomatinae and Leishmaniinae, our analysis supports a clade sister to Leishmaniinae formed by two very-well supported clades: one comprising the genus *Phytomonas* (Phytomonadinae subfamily), whose species parasitize plants and another encompassing the bacterial-symbiont harboring genera*, Strigomonas* and *Angomonas* (Strigomonadinae subfamily). Interestingly, in our study *Herpetomonas muscarum,* which parasitize dipteran flies and *Blechomonas ayalai, a* parasite of fleas, formed a unique subclade sister to Strigomonadinae, a subfamily which includes bacterial endosymbiont harboring trypanosomatids of insects^49–52^. From these previous studies, the genus *Herpetomonas* is more closely related to the genera *Phytomonas* (transmitted to plants by phytophagous hemipterans) and *Lafontella*, the three genera forming the subfamily Phytomonadinae, whereas *Blechomonas ayalai* constituted the monogeneric blechomonadinae^24,51^. To the best of our knowledge, this is the first phylogenomic analysis that includes *Herpetomonas* and *Blechomonas*, whose species are found in closely related orders of insect hosts, Diptera and Siphonaptera, more phylogenetically related between them, than to Hemiptera, the order of the *Phytomonas* vectors^53^. However, phylogenomics, including more species of *Herpetosoma*, and the genus *Lafontella,* are still required to sustain this relationship.

To test the robustness of the method, we generated phylograms using the 25th and 75th percentiles instead of the median 50th percentile (Supplemental Figures S1 and S2, respectively), as well as running the RSD program with 1e−20 instead of 0.001 value for the blast including parameter, and 0.5 instead of 0.8 for the sequence length ratio including parameter (Supplemental Fig. S3). We also randomly reduced the proteomes to one half of their sizes and calculated the resulting median-based phylogram (Supplemental Fig. S4). They are all very similar, with some small deviations in the a.u. values, and *T. vivax* presented shifted placement within *Trypanosoma* cluster, but always with a.u. values smaller than 90.

Many orthology detection strategies are available, which raise many discussions about the ideal ortholog identification method, concerning to sensitivity and specificity. So far none of them is considered a gold standard^54^. The RSD method was applied within the TOMM pipeline because it is the only method which outputs an evolutionary distance measure. The OrthoMCL algorithm has been considered a balanced method identification and its database OrthoMCL-DB is a well-known portal for grouping orthologous protein sequences in a genome-scale across multiple species^55^. However, OrthoMCL does not provide an evolutionary distance measure. Regardless, we checked the RSD-derived orthologs with OrthoMCL-DB via TriTrypDB using the same set of species in which proteins were retrieved from the latter on (Table 1, as indicated in “Protein sequence source” column), in order to enable comparisons between the ortholog lists from obtained RSD and OrthoMCL-DB (Supplemental Table S4). From the total of 78 pairs of species comparison, an average of 87% ± 7.4% (Mean ± SD) of orthologs were detected by both methods. In half of the species combination (39 pairs), the RSD method was able to identify a higher number of orthologs in 16 pairs (20%), representing ortholog pairs exclusively detected by RSD over 50% higher than OrthoMCL-DB (pairwise comparisons with ratio of unique orthologs ≥ 1.5 at column “M” in Supplemental Table S4, e.g*. L. enrietti vs T. evansi* pair #38, which presented 755 unique orthologs with RSD against 446 unique orthologs with OrthoMCL). In turn, 29 pairs of species comparison (37%) represented number of orthologs exclusively detected by OrthoMCL-DB that were over 50% higher than RSD (e.g. pairwise comparisons with ratio of unique orthologs ≤ 0.5 at column “M” in Supplemental Table S4, e.g. *L. tropica vs L. gerbilli* pair #43, which presented 189 unique orthologs with OrthoMCL against 56 unique orthologs with RSD). In the remaining comparisons (43%) the number of unique orthologs detected by each method were homogenous (Supplemental Table S4). Overall, the orthology inference was very dependent for a given pair of species (e.g. *Endotrypanum monterogeii* vs *Leishmania tarentolae* or *Leishmania gerbilli* vs *Trypanosoma evansi)*, but we observed few and homogenous differences between the number orthologs detected by OrthoMCL and RSD, without significant difference between them (Supplemental Fig. S7).

To further test the robustness of our approach, we employed another pipeline for identification of orthologs, using the SonicParanoid^56^ program with the MCL algorithm. This program produces an output with the predicted ortholog pairs from a two species comparison but lacks the calculation of the average amino acid distance between these pairs. We thus wrote a program that generated a fasta file containing the sequences of each ortholog pair, which was submitted to Clustal^57^ alignment, which in turn was submitted to a subroutine of the Mega X package^58^ to calculate the average amino acid distance for the pair. This allowed to generate a SonicParanoid-based amino acid distance matrix that was submitted to Pvclust as described above for the RSD-derived orthologs. The phylogenetic trees of kinetoplastid species generated by the TOMM-RSD and TOMM-SonicParanoid methods can be viewed in Supplemental Figs. S5 and S6. All the branches of the trees depicting the various subgroups are congruent. The main difference between the trees is the location of *Boldo saltans*, which is within the Trypanosomatidae in the RSD-derived tree, with a support of 92%, but in the SonicParanoid tree it is located in between the Leishmanidae and Trypanosomatidae. We conclude that the use of an alternate method of determining the orthologs does not affect the results of the TOMM approach to phylogeny determination. The Sonic approach has the advantage of being very fast compared with the RSD, but the lack of an output of the paired amino acid distances removes this advantage compared to the RSD method. It would be very useful if the sonic paranoid pipeline included the resulting average amino acid distance of the ortholog pairs.

### Hematophagous dipteran tree

The phylogenomic tree for Diptera vectors was built with 29 species from Brachycera (Tsetse flies, *Glossina*) and Nematocera (the majority are *Anopheles* mosquitoes) suborders (Table 2), using the non-hematophagous *D. melanogaster* as outgroup and *M. domestica* as a comparator species for *Glossina* genus. Here, the main vectors related to Kinetoplastid parasites are species from the *Glossina* genus and Psychodidae family (sandflies), which transmit, respectively, African *Trypanosoma* and *Leishmania* protozoans. Hematophagous hemipterans from the subfamily Triatominae are another important group of vectors for *Trypanosoma* parasites; however they were not considered here, because of the high distance in phylogenetic relationship between the Diptera and Hemiptera orders. In fact, due to the great diversity of insects, even inside the Diptera order, it is observed as a very large distance among the families. Such diversity can be verified by the wide range in genome sizes and number of protein-coding genes shown in Table 2.

**Table 2:** Characteristics of genome-deduced proteomes (all* from VectorBase, www.vectorbase.org ) from hematophagous Diptera insects used in this work.

Species	Order level	Family	Common name	Disease’s vector	Geneset version	Genome size (Mb)	Total number of sequences
*Aedes aegypti*	Nematocera, Culicomorpha	Culicidae	Yellow fever mosquito	Dengue, yellow fever, chikungunya and Zika (all viruses)	AaegL5.1	1278	16,355
*Aedes albopictus*	Nematocera, Culicomorpha	Culicidae	Asian tiger mosquito	Dengue, La Crosse encephalitis and West Nile fever	AaloF1.2	1923	15,564
*Anopheles albimanus*	Nematocera, Culicomorpha	Culicidae	American Malaria mosquito	Malaria (*Plasmodium* protozoan)	AalbS2.5	173	11,882
*Anopheles arabiensis*	Nematocera, Culicomorpha	Culicidae	African Malaria mosquito	Malaria (*Plasmodium* protozoan)	AaraD1.8	247	13,221
*Anopheles atroparvus*	Nematocera, Culicomorpha	Culicidae	European Malaria mosquito	Malaria (*Plasmodium* protozoan)	AatrE2.1	225	13,717
*Anopheles christyi*	Nematocera, Culicomorpha	Culicidae	Mosquito	None; comparator species for A. gambiae complex	AchrA1.6	173	10,696
*Anopheles coluzzii*	Nematocera, Culicomorpha	Culicidae	African Malaria mosquito	Malaria (*Plasmodium* protozoan)	AcolM1.6	225	14,502
*Anopheles culicifacies*	Nematocera, Culicomorpha	Culicidae	Asian Malaria mosquito	Malaria (Plasmodium; Apicomplexa protozoan)	AculA1.5	203	14,138
*Anopheles darlingi*	Nematocera, Culicomorpha	Culicidae	American Malaria mosquito	Malaria (*Plasmodium* protozoan)	AdarC3.7	137	10,493
*Anopheles dirus*	Nematocera, Culicomorpha	Culicidae	Asian Malaria mosquito	Malaria (*Plasmodium* protozoan)	AdirW1.7	216	12,711
*Anopheles epiroticus*	Nematocera, Culicomorpha	Culicidae	Asian Malaria mosquito	Malaria (*Plasmodium* protozoan)	AepiE1.6	223	11,854
*Anopheles farauti*	Nematocera, Culicomorpha	Culicidae	Asian/Oceania Malaria mosquito	Malaria (*Plasmodium* protozoan)	AfarF2.4	172	12,967
*Anopheles funestus*	Nematocera, Culicomorpha	Culicidae	African Malaria mosquito	Malaria (*Plasmodium* protozoan)	AfunF1.8	225	13,163
*Anopheles gambiae*	Nematocera, Culicomorpha	Culicidae	African Malaria mosquito	Malaria (*Plasmodium* protozoan)	AgamP4.9	251	13,474
*Anopheles maculatus*	Nematocera, Culicomorpha	Culicidae	Asian Malaria mosquito	Malaria (*Plasmodium* protozoan)	AmacM1.5	302	14,828
*Anopheles melas*	Nematocera, Culicomorpha	Culicidae	African Malaria mosquito	Malaria (*Plasmodium* protozoan)	AmelC2.5	224	14,738
*Anopheles merus*	Nematocera, Culicomorpha	Culicidae	African Malaria mosquito	Malaria (*Plasmodium* protozoan)	AmerM2.7	288	13,264
*Anopheles minimus*	Nematocera, Culicomorpha	Culicidae	Asian Malaria mosquito	Malaria (*Plasmodium* protozoan)	AminM1.7	202	12,455
*Anopheles quadriannulatus*	Nematocera, Culicomorpha	Culicidae	African Malaria mosquito	Malaria (*Plasmodium* protozoan)	AquaS1.9	283	13,168
*Anopheles sinensis*	Nematocera, Culicomorpha	Culicidae	Asian Malaria mosquito	Malaria (*Plasmodium* protozoan)	AsinC2.2	298	19,247
*Anopheles stephensi*	Nematocera, Culicomorpha	Culicidae	Asian Malaria mosquito	Malaria (*Plasmodium* protozoan)	AsteI2.3	223	11,699
*Culex quinquefasciatus*	Nematocera, Culicomorpha	Culicidae	Southern house mosquito	lymphatic filariasis (worm), West Nile fever and St. Louis encephalitis (viruses)	CpipJ2.4	579	18,364
*Drosophila melanogaster*1	Brachycera, Muscomorpha	Drosophilidae	Fruit fly	None; comparator species for dipterans	–	138	17,261
*Glossina austeni*	Brachycera, Muscomorpha	Glossinidae	Tsetse fly	Animal African Trypanosomiasis (*Trypanosoma* protozoan)	GausT1.6	370	19,732
*Glossina brevipalpis*	Brachycera, Muscomorpha	Glossinidae	Tsetse fly	Animal African Trypanosomiasis (*Trypanosoma* protozoan)	GbreI1.6	315	14,650
*Glossina fuscipes*	Brachycera, Muscomorpha	Glossinidae	Tsetse fly	Human African Trypanosomiasis (*Trypanosoma* protozoan)	GfusI1.6	375	20,141
*Glossina morsitans*	Brachycera, Muscomorpha	Glossinidae	Tsetse fly	Human and Animal African Trypanosomiasis (*Trypanosoma* protozoan)	GmorY1.9	355	12,507
*Glossina pallidipes*	Brachycera, Muscomorpha	Glossinidae	Tsetse fly	Human African Trypanosomiasis (*Trypanosoma* protozoan)	GpalI1.6	357	19,308
*Lutzomyia longipalpis*	Nematocera, Psychodomorpha	Psychodidae	Sand fly	American Visceral Leishmaniasis (*Leishmania* protozoan)	LlonJ1.4	154	10,284
*Musca domestica*	Brachycera, Muscomorpha	Muscidae	House fly	None; comparator species for Glossina	MdomA1.3	636	15,116
*Phlebotomus papatasi*	Nematocera, Psychodomorpha	Psychodidae	Sand fly	Old World cutaneous Leishmaniasis (*Leishmania* protozoan)	PpapI1.4	364	11,152

*Except for *Drosophila*; 1. Obtained from NCBI.

The phylogram for hematophagous dipterans was based on an average of 8168 orthologous proteins, with a minimum number of ortholog sequences (5893) found in *Anopheles maculatus/ Lutzomyia longipalpis* pair of vectors species. The highest number of2 ortholog sequences was 13,161 between the Tsetse flies *Glossina austeni* and *Glossina pallipides* (Supplemental Table ). To the best of our knowledge, these are the highest numbers of orthologous genes considered for taxa inside Diptera, as collectively surveyed previously^59^.

The well-known *D. melanogaster* was considered an outgroup species to hematophagous dipterans, but as a dipteran it has not presented a proper isolation of an outgroup, being positioned inside the highly supported Brachycera clade (a.u. 100). In general, as observed for Primates and Kinetoplastida, the TOMM approach was also robust in building the phylogenetic relationships to this group of dipterans (Fig. 3). The Nematocera clade presented a moderate support (a.u. 73), which can be explained by a split in two families, Psychodidae (*Lutzomyia longipalpis* and *Phlebotomus papatasi*) and Culicidae (*Anopheles*, *Aede*s and *Culex*) (Fig. 3). Interestingly, in previous insect phylogenomics studies, Culicidae species have been placed apart from all other dipterans, and although more externally, Psychodidae is positioned in the same clade with *Glossina* and *Drosophila*^54^. However, here we have found an opposite topology reached by TOMM phylogram for Psychodidae species, in which *Phlebotomus* and *Lutzomyia* were more closely related to Culicidae (all Nematocera) than the Brachycera species (*Glossina* genus).

The evolutionary relationships of Anopheline mosquitoes are widely studied because of the great medical importance of this group as vectors of Malaria, especially the *Anopheles gambiae* complex, which is composed of eight species morphologically indistinguishable; however the species display differential traits such as, behavior, ecological niche, and vector competence^60^. Using whole-genome reference sequences, different phylogenetic relationships between genomic regions have been inferred for *A. gambiae* complex when differential analyses target autosomes or sex chromosomes and coding or non-coding loci^60,61^. A consensus phylogenetic relationship between *A. gambiae* (G) and *A. coluzzi* (C) as a sister group (G + C) was found in two comprehensive studies using X chromosome or autosomes, employing Maximum-Likelihood- (ML)^60^ or Bayesian Multispecies Coalescent model-^61^ based methods. In addition, another sister group composed of *A. arabiensis* (A) and *A. quadrianulatus* (Q) was inferred only when X chromosome genomic regions were used^60,61^.

Here, the clade topology of *A. gambiae* complex reached by TOMM approach (Fig. 3) corroborates the sister group A + Q inferred by known X chromosome phylogenies with high confidence (a.u. 98). However, the topology for other species relationships depicted a different scenario. Of note, G + C were not placed together in a same branch and *A. merus* (R), often branched in a more external position of the trees, was significantly (a.u. 96) placed more internally close to A + Q pair. Moreover, *A. melas* (L) was the earliest branched species in the clade; whereas in known phylogenies, *A. merus* was placed in this position. Thus, while the most recent topologies^61^ for *A. gambiae* complex presented patterns as (R((L(A + Q))(G + C))) for non-coding and ((L(A + Q))(R(G + C))) for coding data from X chromosome, the TOMM approach reassembled the pattern (L(G(C(R(A + Q))))) using all sets of orthologous proteins (over 8000 coding sequences) found for the 29 species used.

### Primates tree

The Primates phylogenomic tree included 25 species presenting published whole-genome sequence, encompassing all sublevels of the order, including lemurs, lorises, tarsiers, New World Monkeys (NWM), Old World Monkeys (OWM), big apes, and humans^15^, and includes the two additional mammals species that were used as outgroups (see Table 3). The Primates phylogram was based on aveage 23,826 orthologous proteins, with a minimum number of ortholog sequences (19,185) found in *Propithecus coquereli*/*Carlito syrichta* pair of primate species. If considered the entire phylogram, which includes the two outgroups species, the overall minimum number of ortholog sequences was 18,970 (*Tupaia chinensis/Carlito syrichta* pair). The highest number of ortholog sequences was 39,341 between *Homo sapiens* and *Pan troglodytes*, showing that the topology achieved by the TOMM approach accounts for both the number of orthologs, as well as amino acid distances (Supplemental Table 3).

**Table 3:** Characteristics of genome-deduced proteomes (all from NCBI*) from mammals used in this work.

Species	Order levels	Family	Abbreviation	Common name	Genome size (Mb)	Total number of sequences1
*Aotus nancymaae*	Simiformes, Platyrrhini	Aotidae	AOTNAN	Ma's night monkey	2862	30,849
*Callithrix jacchus*	Simiformes, Platyrrhini	Cebidae	CALJAC	White-tufted-ear marmoset	2733	31,373
*Carlito syrichta*	Tarsiiformes	Tarsiidae	CARSYR	Philippine tarsier	3454	26,764
*Cebus capucinus*	Simiformes, Platyrrhini	Cebidae	CEBCAP	White-faced sapajou	2718	35,515
*Cercocebus atys*	Simiformes, Catarrhini	Cercopithecidae	CERATY	Sooty mangabey	2848	38,743
*Chlorocebus sabaeus*	Simiformes, Catarrhini	Cercopithecidae	CHLSAB	Green monkey	2790	38,532
*Colobus angolensis*	Simiformes, Catarrhini	Cercopithecidae	COLANG	Angolan colobus	2970	28,757
*Gorilla gorilla*	Simiformes, Catarrhini	Hominidae	GORGOR	Western gorilla	3074	31,611
*Homo sapiens*	Simiformes, Catarrhini	Hominidae	HOMSAP	Human	3096	54,793
*Macaca fascicularis*	Simiformes, Catarrhini	Cercopithecidae	MACFAS	Crab-eating macaque	2947	36,852
*Macaca mulatta*	Simiformes, Catarrhini	Cercopithecidae	MACMUL	Rhesus monkey	3097	34,238
*Macaca nemestrina*	Simiformes, Catarrhini	Cercopithecidae	MACNEM	Pig-tailed macaque	2949	37,815
*Mandrillus leucophaeus*	Simiformes, Catarrhini	Cercopithecidae	MANLEU	Drill	3062	28,631
*Microcebus murinus*	Lemuriformes	Cheirogaleidae	MICMUR	Gray mouse lemur	2487	33,966
*Nomascus leucogenys*	Simiformes, Catarrhini	Hylobatidae	NOMLEU	White-cheeked gibbon	2962	28,771
*Otolemur garnettii*	Lorisiformes	Galagidae	OTOGAR	Small-eared galago, or Bushbaby	2520	25,278
*Pan paniscus*	Simiformes, Catarrhini	Hominidae	PANPAN	Pigmey chimpanzee	3287	31,623
*Pan troglodytes*	Simiformes, Catarrhini	Hominidae	PANTRO	Chimpamzee	2892	45,468
*Papio anubis*	Simiformes, Catarrhini	Cercopithecidae	PAPANU	Olive baboon	2959	39,065
*Piliocolobus tephrosceles*	Simiformes, Catarrhini	Cercopithecidae	PILTEP	Ugandan red Colobus	2923	33,549
*Pongo abelli*	Simiformes, Catarrhini	Hominidae	PONABE	Sumatran orangutan	3253	32,655
*Propithecus coquereli*	Lemuriformes	Indiidae	PROCOC	Coquerel's sifaka	2798	23,684
*Rhinopithecus bieti*	Simiformes, Catarrhini	Cercopithecidae	RHIBLE	Black snub-nosed Monkey	2977	32,121
*Rhinopithecus roxellana*	Simiformes, Catarrhini	Cercopithecidae	RHIROX	Golden snub-nosed monkey	2900	28,672
*Saimiri boliviensis boliviensis*	Simiformes, Platyrrhini	Cebidae	SAIBOL	Bolivian squirrel monkey	2609	26,794
*Tupaia chinensis* (outgroup)	Euarchontoglires, Scadentia	Tupailidae	TUPCHI	Chinese tree shrew	2847	27,162
*Mus musculus* (outgroup)	Euarchontoglires, Rodentia	Muridae	MUSMUS	Common mouse	2654	76,190

* https://www.ncbi.nlm.nih.gov/genome ; protein sequences were retrieved from RefSeq database.

1. After clusterization at 99% and removal of sequences smaller than 50 aa.

The Primates phylogram showed correctly *Mus musculus* as an outgroup and several well-formed clades within the Strepsirrhini and Haplorrhini suborders (Fig. 4). Main taxonomic groups at suborder sublevels (Catarrhini and Platyrrhini), as well as at family level (Cercopithecidae and Hominidae), resemble current knowledge (Lockwood et al. 2004; Langergraber et al. 2012; Freitas et al. 2018). Among the superfamily Hominoidae, the human location and its relationship with the gorilla and chimpanzee/bonobo clades (a.u. 100) from the Homininae subfamily was similar to that shown in previous studies^62–64^, suggesting an accelerated evolution of human genes, as proposed by Hubisz and Pollard^65^. The position of *Nomascus leucogenys,* the critically endangered gibbon from the Hylobatidae family, is also accurate^66^. However, two clades showed different clustering compared to other Primates phylogenomic studies^18,23^: one regarding OWM (Catarrhini) from Cercopithecoidea (highlighted in blue) and another clustering NWM (Plathyrrhini) from *Aotus* and *Cebus* genres (highlighted in red) (Fig. 4).

*Cercocebus atys* is an OWM, who inhabits the West African forests (from Senegal and Congo), considered, by IUCN, as Vulnerable (VU)^66^. This species is naturally infected by the Simian Immunodeficiency Virus (SIVsmm), and due to its close-relationship with humans, the hazardous form of this virus, HIV-2 (Human Immunodeficiency Virus, type 2), was transmitted to man^67^. Such genus has been commonly placed closer to the baboons from *Mandrillus* genus^68,69^. However, we did not use any protein collection from *Mandrillus* species in our approach. The most related species from Papionini tribe used herein was from *Macaca* genus and from the widest-ranging baboon *Papio anubis*, which clustered with *C. atys,* and then to *Macaca* species, that showed highly supported clades*.*

Related to the NWM platyrrines, *Cebus capucinus* from the Cebidae family clustered with the only night monkey species with complete genome sequence available, *Aotus nancymaae* from the Aotidae family, rather than the other Cebidae representative, *Saimiri boliviensis* (Fig. 4). *Aotus* neotropical monkeys are often used as a primate biological model for *Plasmodium* infection in Malaria researches^70^, raising extensive discussions about their evolutionary relationships with other NWM^71^. Classical overviews on adaptive radiation of neotropical primates, discussing phylogenetic relationships and inconsistences among *Saimiri*, *Cebus* and *Aotus*, highlighted discordances between morphological and molecular analyses^72,73^. Nevertheless, mostly molecular approaches have usually considered *Saimiri* and *Cebus* as representatives from the Cebidae family, and *Aotus* as a distinct clade from Aotidae^63,64,73^. Such results were also observed by the most complete primate mitogenomics performed to date^17^. Our TOMM phylogenomic tree revealed a low probability supported clade (a.u. 55), clustering *Aotus* and *Cebus* when a cutoff value of 50% was considered. Such unresolved clustering may have been shaped by influence of the total number of orthologous proteins found among the three species, since *Aotus-Cebus* pair presented more orthologous proteins (25,629), than *Saimiri-Cebus* (24,085) or *Aotus-Saimiri* (23,205) (Supplemental Table 3). Thus, the results presented here should maintain this evolutionary debate within the field of primatology.

## Concluding remarks

Even with genomic data available for several groups of organisms along the tree of life, reaching a definitive evolutionary relationship among taxa is still hard. That is because evolution of genomes undergoes great dynamic evolutionary processes with different pressures depending on the genomic region and gene product function. Evaluating phylogenomic relationships depends on numerous supervised methods and procedures, all subject to variable benefits and disadvantages, where a trade-off between accuracy and objectivity is pondered relying on the type of application. Despite all these caveats, there is no hesitation that Phylogenomics is a powerful integrated field that is raising key questions in the evolutionary history of several group of organisms and providing very useful information, whether for biodiversity conservation or in agriculture, livestock, and biomedical matters.

Here, we presented the TOMM approach for phylogenomic analysis, which uses genome-wide protein-coding sequences for a given group of organisms, gathering orthologous predicted proteomes between pairs of desired taxa in order to build a single phylogram based on their median amino acid evolutionary distances. This unsupervised approach was basically divided in two extensive steps, where the first consists of orthology inference and the second is composed of steps to build a large pairwise amino acid distance matrix; this latter is the novelty along the rational analysis for Phylogenomics.

Regarding the first step, as any other phylogenetic analysis, TOMM approach relies on inferring orthologs. Reliable orthologs identification between genome sequences is challenged by how different evolutionary mechanisms operate in different genomic regions. As surveyed and discussed elsewhere^74^, there are several methods for orthology inference, all presenting advantages and limitations, but the most common methods are based on sequence similarity. Here, we used Reciprocal Smallest Distance (RSD) method^75^, which is obtained from sequence similarity metrics within an evolutionary distance matrix. Also, RSD uniquely provides an amino acid distance measure. Many different orthology inference methods were not evaluated during TOMM approach, because our aim was not to test orthology detection performance, rather to perform a comprehensive phylogenomic analysis based on all pairwise orthologous pairs found inside a group.

Since there is no choice of gene families or genomic regions, as many phylogenomic studies ascribed them, we denominate our approach as unsupervised and “total”. The originality of our phylogenomic analysis is related to the second step of procedure, through the construction of a species matrix populated with evolutionary distance measurements calculated in the previous step, rather than performing multiple sequence alignments. However, sequence alignments were embedded during orthology detection. We assigned the “median” amino acid distance between two taxa as a measurement to populate the species matrix and then building the phylogram, but by testing other percentiles of distance measures, we observed that the TOMM approach has kept the robustness of results about well-known phylogenetic relationships.

Possible criticisms concerned to our approach are i) the computational resources needed, because the RSD method is computationally intensive, and it worsens for large genomes and ii) the customized programs to help building the amino acid distance matrix are operational system-restricted (Windows Microsoft). The first step is not feasible to common PC machines and it must be performed within HPC resources. However, with the increasing availability of HPCs whether offered by public or private institutions and virtual machines as emulators of computational systems, make these two concerns minor caveats. Another concern is related to sampling taxa; the benefit of use the total predicted proteomes has a limitation in the number of publicly available organisms with annotated genome sequences. Even though, we showed here that TOMM approach is applicable and robust for wide range of taxa presenting distinct genome sizes and complexity, since we applied to Kinetoplastid (9.5–58.7 Mb haploid genome size), hematophagous Diptera (137–1923 Mb haploid genome size), and Primates (2487–3454 Mb haploid genome size). Its robustness was also verified when trees were generated from genomes reduced randomly to 50% of their sizes, when very similar trees were obtained (Supplemental Fig. S4).

Finally, this approach was not only able to corroborate the main knowledge in phylogenetic relationships of tested groups of organisms, but also to present novel branch topologies. We believe that our results with TOMM should contribute to supporting and enriching the evolutionary insights to the field.

## Methods

### Sequence datasets

We used protein sequences of all protein-coding genes (proteome), deduced from a complete genome for a given species, downloading data from Kinetoplasdida, Diptera, and Primates, as well as other external organisms (Table 1, 2, 3).

The Kinetoplastid genomic sequences from 46 species were downloaded from NCBI (https://www.ncbi.nlm.nih.gov/genome) or TriTrypDB (http://tritrypdb.org/tritrypdb/) databases, according to information provided in Table 1. The protein sequences corresponding to coding sequences from a given Kinetoplastid genome were downloaded when available. When protein sequences were not available, as the genes of these organisms do not contain introns, we straightforwardly translated them *in-house* from genomic sequences by obtaining open reading frames from six the translations using the EMBOSS tool^76^ and adjusting the starting Methionine by BLASTX to the Protozoa-RefSeq NCBI database. This information was specified in Table 1 at “protein sequence source” column. *Perkinsela sp.* was used as outgroup.

For the hematophagous dipterans dataset, all protein sequences were downloaded from VectorBase^77^ (https://www.vectorbase.org/downloads) as specified in Table 2, except for *Drosophila melanogaster*, which was downloaded from RefSeq NCBI database. Both the non-hematophagous flies, *Musca domestica* and *D. melanogaster.* were used as related species.

For the Primates, we used annotated complete genomes of 25 species, including *Homo sapiens*. *Mus musculus* (House mouse) and *Tupaia chinensis* (Chinese tree shrew) were used as an outgroup. All protein sequences of the mammalians were downloaded from RefSeq Protein NCBI database (https://www.ncbi.nlm.nih.gov/refseq/) (Table 3).

### Data analyses

The TOMM pipeline was performed in several steps, as shown in Fig. 1. First, the protein sequences were dereplicated, and then clustered at 99% identity. The centroids were saved using the Usearch program version 9.0^78^. Only downloaded protein sequences or translated protein-coding genomic sequences larger than 50 amino acids were used in the subsequent analyses. To sample the proteome to 50% of its level, we used the program Seqtk available at https://github.com/lh3/seqtk. Second, the proteomes from each of the downloaded genomes (or translated coding sequences *in-house*) were pairwise submitted to the program Reciprocal Smallest Distance (RSD)^75^ to obtain a table of orthologs and their amino acid (aa) distances. The RSD algorithm employs global sequence alignment by using ClustalW^79^ and maximum likelihood by using PAML^80^ to estimate the amino acid substitutions. To build the matrix of median pairwise amino acid distances (AAD) from genome-derived protein sequences, pairs of proteomes [the number of pairs is equal to (n2 – n)/2, where n = number of species], for each taxonomic group used here, were submitted to the program RSD using the NIH Biowulf cluster (https://hpc.nih.gov/systems/). For the Kinetoplastida and hematophagous Diptera, we used the RSD settings of 0.001 for the blast e-value of acceptance, and the value of 0.8 for the minimum ratio of the smallest sequence to the larger one. For Primates, the e-value of acceptance was 0.1. The RSD tables were sorted by their AAD’s to obtain the desired percentile values of AAD. Matrices were constructed for specified percentile values. These matrices were then submitted to the Hclust^81^ and Pvclust^82^ packages into R version 3.5.2^83^ to obtain phylograms, after 10,000 bootstraps. The APE package^84^ was used to export the trees (in Newick format), and these were annotated using the MEGA 6 software^85^. The approximately unbiased values of the nodes (expressed as α values, where α = 1- P), as provided by Pvclust, were exported to a Newick file by modifying a function provided at https://stackoverflow.com/questions/22749634/how-to-append-bootstrapped-values-of-clusters-tree-nodes-in-newick-format-in. The R script for these operations is shown in Supplemental File 1.

To compare the orthologs identified by RSD with those inferred by MCL algorithm, we used SonicParanoid. A *in-house* script compiled the protein sequences of each ortholog pair in a fasta file, which in turn was submitted to multiple sequence alignment (MSA) using Clustal^57^. Then, the amino acid divergence was calculated using the MSA in a routine of the the Mega X package^58^, resulting in a SonicParanoid-based amino acid distance matrix.

To compare the orthologs detected by RSD with those of the TriTypDB database, searches were performed using the TriTypDB database (https://tritrypdb.org). For this, all genes of the species *Endotrypanum monterogeii, Leishmania aethiopica, Leishmania arabica, Leishmania enriettii, Leishmania tropica, Leishmania gerbilli, Leishmania turanica, Leishmania tarentolae, Trypanosoma evansi, Trypanosoma vivax, Trypanosoma theileri, Blechomonas ayalai* and *Crithidia fasciculata* were compared in pairs using “Identify Genes based on Orthology Phylogenetic Profile” tool, determined by the OrthoMCL algorithm^86^ under OrthoMCL-DB, yielding 78 pairwise comparisons. For method comparison, intersections between RSD-derived and OrthoMCL-derived orthologs were calculated using respective gene ID lists as input in custom Venn diagram tool available at http://bioinformatics.psb.ugent.be/webtools/Venn/.

### Customized In-House programs to retrieve orthologous sequence from RSD

Three programs were written in Visual Basic v6.0 to facilitate the step of orthologous identification in the pipeline. These are named RSD-maker, Batcher, and RSD2Table. They are available for download at https://s3.amazonaws.com/proj-bip-prod-publicread/transcriptome/Tomm/Tomm-executables.zip.

RSD-maker takes as input a list of FASTA file names and produces a tab-delimited list of all pairs of FASTA files to be submitted to RSD. It can take also an additional list of FASTA pairs already processed, and in this case, it outputs only the missing pairs. This is useful when an additional proteome is added after RSD has been run on a group of sequences. The sequence pairs for each pairwise RSD comparison are then provided as input to the program Batcher, which also takes as input the command line for the RSD program, such as “rsd_search -q INPUT1 -subject-genome = INPUT2 -outfmt 1 -de 0.8 0.1 -o output/INPUT1-INPUT2-0.8_0.1.tbl”. Upon running the program, INPUT1 and INPUT2 will be substituted by the tab-delimited pair to produce a file containing hundreds or thousands of commands as dictated by the number of pairs used as input (RSD resulting files). Such resulting file is used to run simultaneously as a swarm in the NIH Biowulf HPC (High-Performance Computing; http://hpc.nih.gov). The RSD resulting files (Supplemental File 2, as compressed folders “RSD-Primates”, “RSD-Flies”, RSD-kinetoplastids”) contain gene ID lists tabulated for INPUT1 species (first column) and INPUT2 (second column), they are then processed by the program RSD2Table. It takes as input the list of FASTA files as well as a list of the RSD results, and the desired percentile value. It then sorts the RSD files in ascending order of the AAD values and finds the AAD corresponding to the desired percentile. This program can also receive a list of desired percentiles and then produces all matrices in a single run. In addition to the aa distance matrix of the orthologs, it also produces a table indicating the number of ortholog pairs found by RSD. The matrices are written as “table-10.tbl” or “table-50.tbl”, where 10 and 50 are the pre-determined percentiles. These matrices can then be submitted to the program Batcher, that will take as INPUT1 the list of percentiles and the R script shown in Supplemental File 1, to produce an output that can be pasted on the R console to produce the Pvclust results and Newick file as described in the previous paragraph.

The main computationally intensive job for identification of orthologous sequences is the calculation of the RSDs, which can take a few hours per CPU for the smaller Kinetoplastid genomes, to over one day for the larger genomes such as from the Primates. For example, the 27 mammal species used in this work lead to 351 pairwise comparisons, which could consume over one year of computational time for a single CPU. However, no more than 4 GB of memory is needed per CPU, and the job can be easily parallelized on an HPC system, so the results were obtained in approximately two days.

## Supplementary Information

Supplementary Figures.

Supplementary Information 1.

Supplementary Information 2.

Supplementary Information 3.

Supplementary Table S1.

Supplementary Table S2.

Supplementary Table S3.

Supplementary Table S4.

## Data Availability

The Supplemental File 2 is a compressed folder available in the Dryad repository at https://doi.org/10.5061/dryad.b1k526g, as well as all other supplemental data supporting the results of this article.
