# Supplementary material for: Total Ortholog Median Matrix as an alternative unsupervised approach for phylogenomics based on evolutionary distance between protein coding genes
Source: Sci Rep. 2021 Feb 15;11:3791. doi: 10.1038/s41598-021-81926-w (PMC7884790; doi:10.1038/s41598-021-81926-w)
Supplement: Supplementary file 1 — Supplementary Figures. [file 41598_2021_81926_MOESM1_ESM.pdf]

Supplemental figure 1 (S1): Phylogram deriving from the 25th percentile of the ranked ortholog pairs of 46 species of kinetoplastids. Abbreviations at the end of the file.

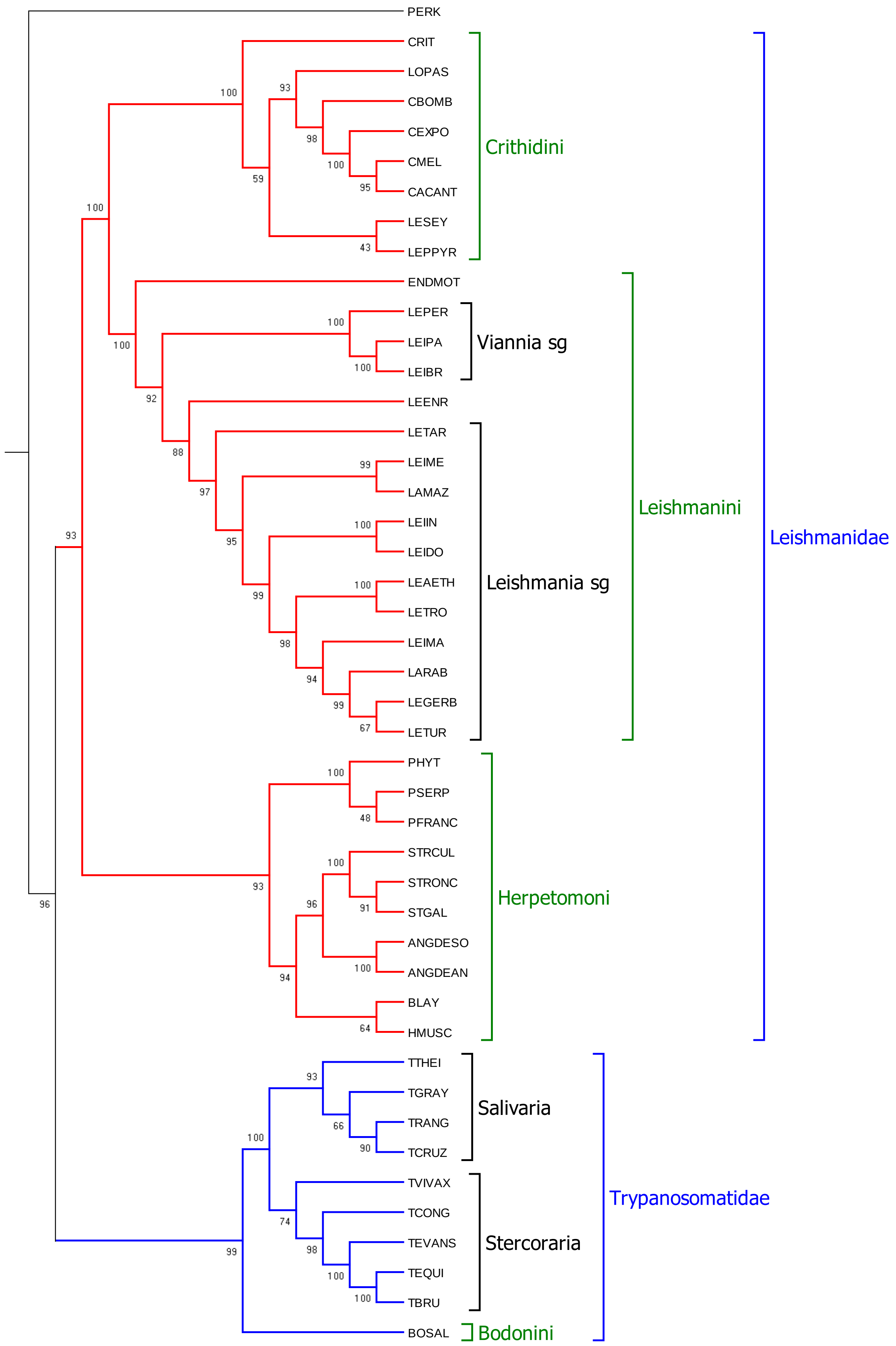

Supplemental figure 2 (S2): Phylogram deriving from the 75th percentile of the ranked ortholog pairs of 46 species of kinetoplastids. Abbreviations at the end of the file.

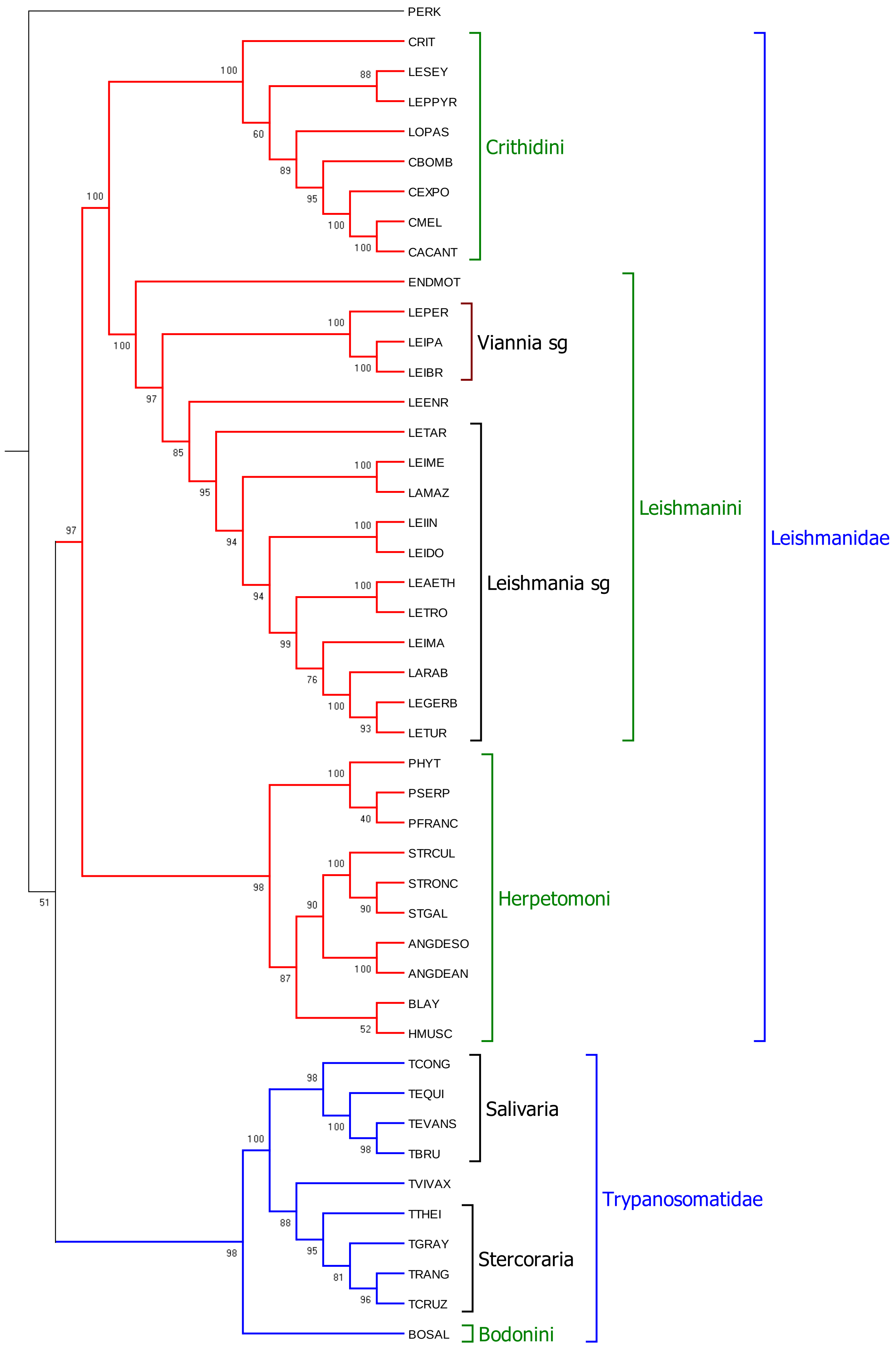

Supplemental figure 3 (S3): Phylogram deriving from the 50th percentile of the ranked ortholog pairs of 46 species of kinetoplastids using RSD parameters of 1e-20 and 0.5. Abbreviations at the end of the file.

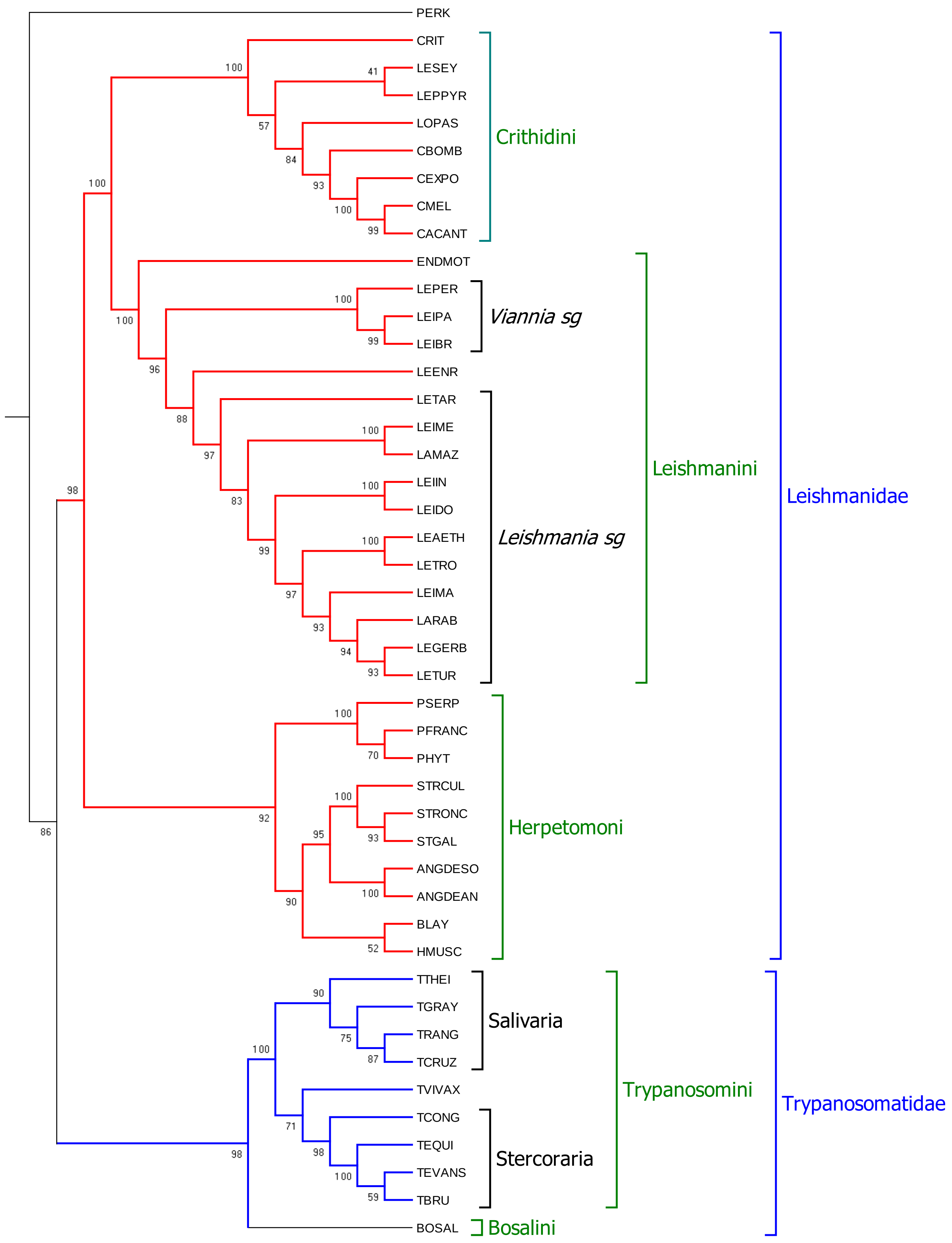

Supplemental figure 4 (S4): Phylogram deriving from the 55th percentile of the ranked ortholog pairs of 46 species of kinetoplastids that had their proteome randomly reduced to one half of its size. Abbreviations at the endo of the file.

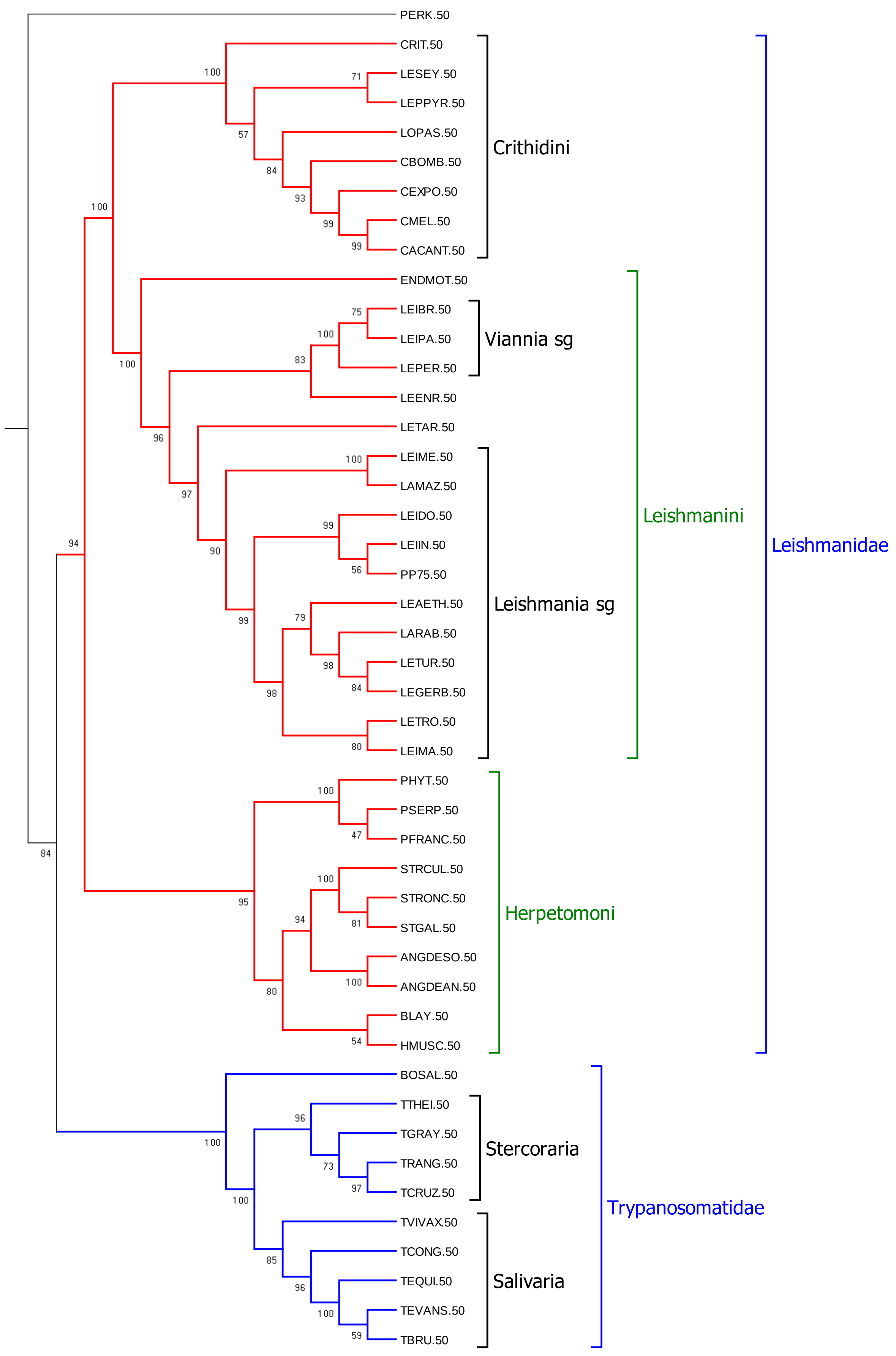

Supplemental figure 5 (S5): Phylograms for Kinetoplastida derived from different methods for orthologs inference: a) RSD, based on evolutionary distance; b) SonicParanoid, based on MCL algorithm. Abbreviations at the end of the file.

a) RSD

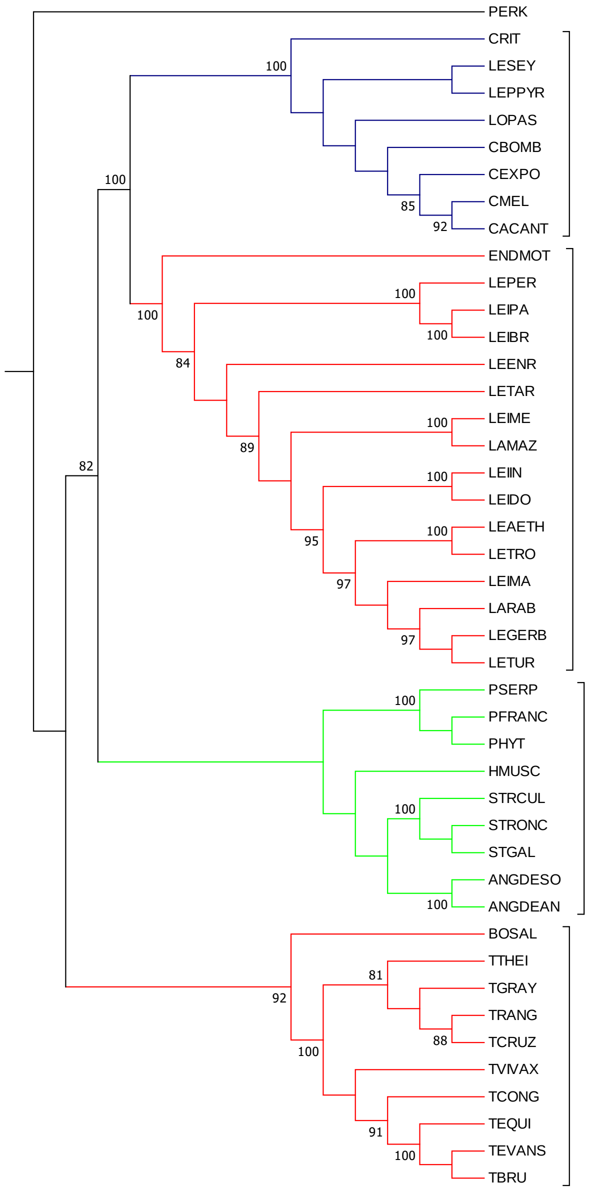

b) SonicParanoid

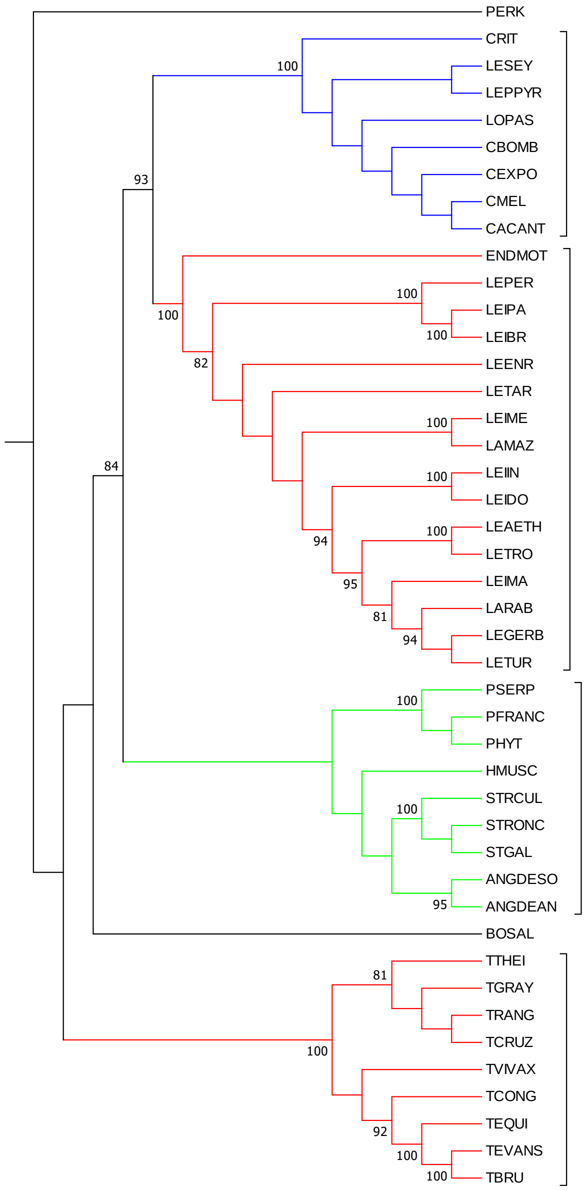

Supplemental figure 6 (S6): Overlapped phylograms for Kinetoplastida derived from different methods for orthologs inference. RSD method in red and SonicParanoid in blue. Abbreviations at the end of the file.

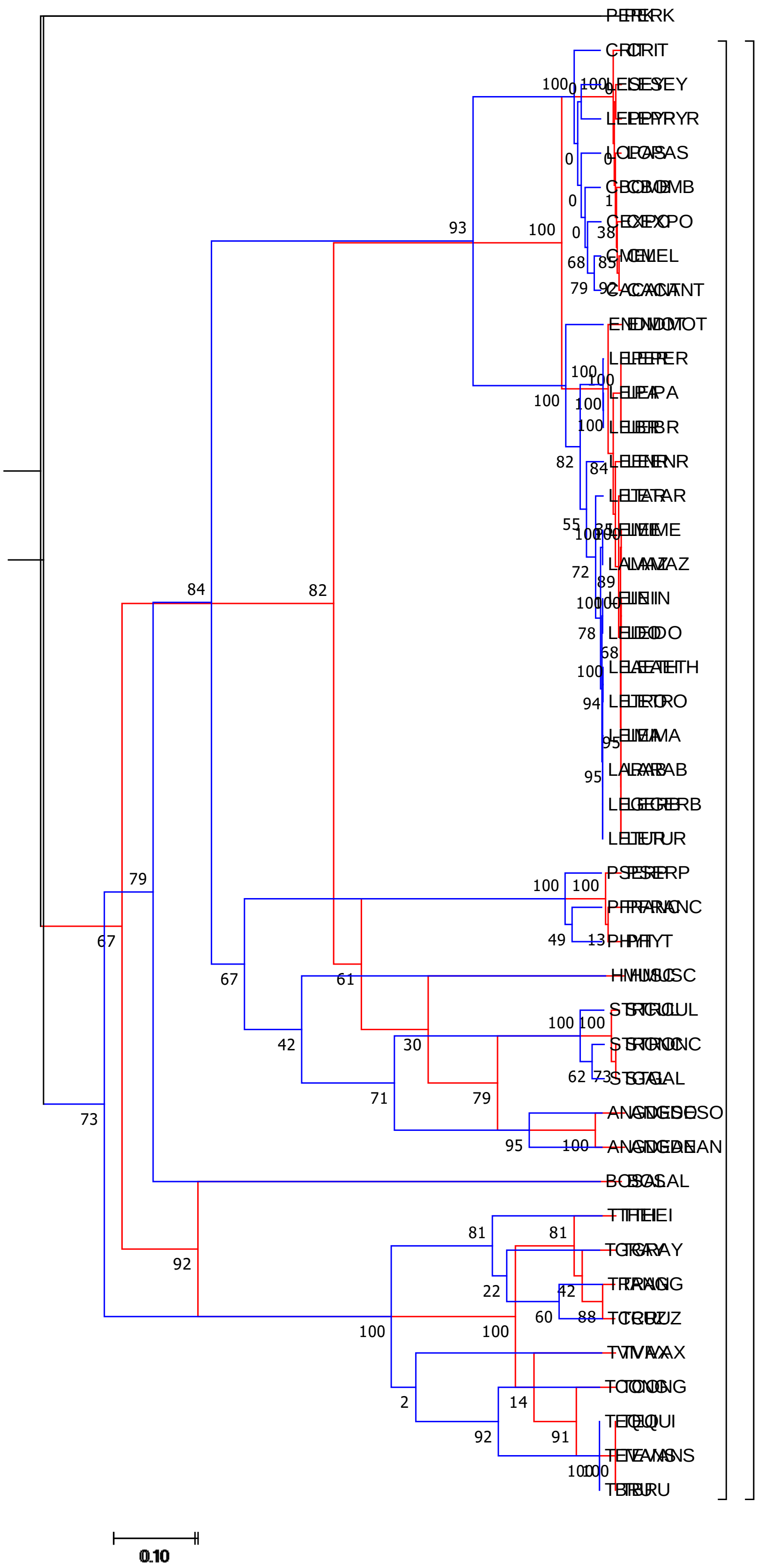

Supplemental figure 7 (S7): Bar graph showing the total number of orthologous identified by the RSD and OrthoMCL algorithms for 78 pairs of species combinations used in the analysis, based on 13 species with sequences retrieved from TriTryp database, as indicated in Table 1 ("Proteins sequence source" column). Intersections (shared orthologs) and unique orthologs were calculated with gene ID lists as input using Venn diagram tool (http://bioinformatics.psb.ugent.be/webtools/Venn/). Blue bars represent the number of orthologous genes identified by both algorithms, whereas red bars indicate the number of unique orthologs identified by each algorithm. Total orthologs for RSD: 7013 ± 831 and OrthoMCL: 7017 ± 977. Unique orthologs detected by RSD: 457 ± 36 and OrthoMCL: 556 ± 34. Values represent mean ± SEM.

File name: Supplemental_Figures_S1_S2_S3_S4_S5_S6_S7.pdf

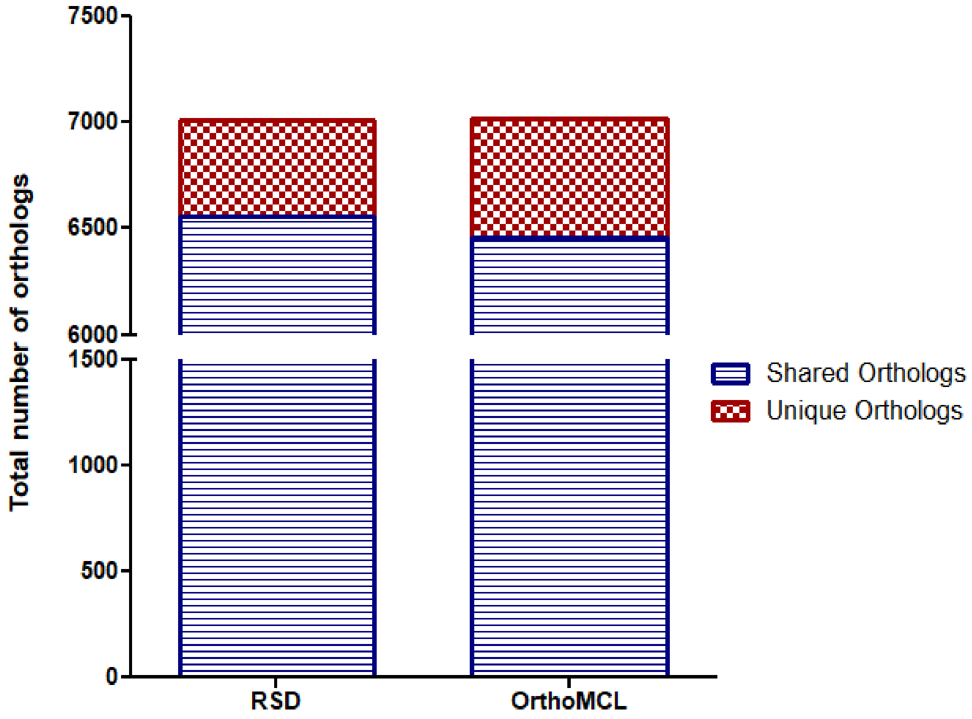

Table of abbreviations. The Kinetoplastida species names used in supplemental figures from S1 to S6 are abbreviated as follows:

**Species name**	**Abbreviation**
Angomonas deanei	ANGDEAN
Angomonas desouzai	ANGDESO
Blechomonas ayalai	BLAY
Bodo saltans	BOSAL
Crithidia acanthocephali	CACANT
Crithidia bombi	CBOMB
Crithidia expoeki	CEXPO
Crithidia mellificae	CMEL
Crithidia fasciculata	CRIT
Endotrypanum monterogeii	ENDMOT
Herpetomonas muscarum	HMUSC
Leishmania amazonensis	LAMAZ
Leishmania arabica	LARAB
Leishmania aethiopica	LEAETH
Leishmania enriettii	LEENR
Leishmania gerbilli	LEGERB
Leishmania braziliensis	LEIBR
Leishmania donovani	LEIDO
Leishmania infantum	LEIIN
Leishmania major	LEIMA
Leishmania mexicana	LEIME
Leishmania panamensis	LEIPA
Leisnmania peruviana	LEPER
Leptomonas pyrrhocoris	LEPPYR
Leptomonas seymouri	LESEY
Leishmania tarentola	LETAR
Leishmania tropica	LETRO
Leishmania turanica	LETUR
Trypanosoma evansi	LEVANS
Lotmaria passim	LOPAS
Perkinsela sp.	PERK
Phytomonas francai	PFRANC
Phytomonas sp.	PHYT
Phytomonas serpens	PSERP
Strigomonas galati	STGAL
Strigomonas culicis	STRCUL
Strigomonas oncopelti	STRONC
Trypanosoma brucei	TBRU
Trypanosoma congolense	TCONG
Trypanosoma cruzi	TCRUZ
Trypanosoma equiperdum	TEQUI
Trypanosoma grayi	TGRAY
Trypanosoma rangeli	TRANG
Trypanosoma theileri	TTHEI
Trypanosoma vivax	TVIVAX
