# Supplementary material for: Total Ortholog Median Matrix as an alternative unsupervised approach for phylogenomics based on evolutionary distance between protein coding genes
Source: Sci Rep. 2021 Feb 15;11:3791. doi: 10.1038/s41598-021-81926-w (PMC7884790; doi:10.1038/s41598-021-81926-w)
Supplement: Supplementary file 2 — Supplementary Information 1. [file 41598_2021_81926_MOESM2_ESM.pdf]

```
library(pvclust)
library(snow)
library(ape)
# change to your working directory below
setwd("c:/rsd")
rm(fit)
rm(x)
# Change INPUT1 found several times below for the name of the
matrix table to be analysed
data <- read.delim("table-
INPUT1.tbl",sep="\t",header=TRUE,dec=".",stringsAsFactors =
FALSE,strip.white = TRUE)
x <- as.matrix(data)
fit <- pvclust(x,
method.hclust="average",parallel=TRUE,nboot=10000)
plot(fit, main="PVCLUST with AU values - ortholog percentile =
INPUT1 %", col.pv=c(2,0,0), cex.pv=0.6, float=0.015)
pvrect(fit, alpha=0.95, pv="au", type="geq", max.only=FALSE)
#saves plot generated by pvclust in wmf format
savePlot(filename="INPUT1-pvclust",type=c("wmf"),
device=dev.cur())

#below saves clusters of species found
sink(file="clusters-INPUT1.txt")
pvpick(fit, alpha=0.95, pv="au", type="geq", max.only=FALSE)
sink()

#the function below saves the a.u. values to the newick file
as.phylo.hclust.with.nodenames <- function (x, nodenames, ...)
#We add a nodenames argument
{
    N <- dim(x$merge)[1]
    edge <- matrix(0L, 2 * N, 2)
    edge.length <- numeric(2 * N)
    node <- integer(N)
    node[N] <- N + 2L
    cur.nod <- N + 3L
    j <- 1L
    for (i in N:1) {
        edge[j:(j + 1), 1] <- node[i]
        for (l in 1:2) {
            k <- j + l - 1L
            y <- x$merge[i, l]

            if (y > 0) {
                edge[k, 2] <- node[y] <- cur.nod
                cur.nod <- cur.nod + 1L
```

```
                edge.length[k] <- x$height[i] - x$height[y]
            }
            else {
                edge[k, 2] <- -y
                edge.length[k] <- x$height[i]
            }
        }
        j <- j + 2L
    }
    if (is.null(x$labels))
        x$labels <- as.character(1:(N + 1))
    node.lab <- nodenames[order(node)] #Here we define our node
labels
    obj <- list(edge = edge, edge.length = edge.length/2,
tip.label = x$labels,
        Nnode = N, node.label = node.lab) #And you put them in
the final object
    class(obj) <- "phylo"
    reorder(obj)
}


yy<-as.phylo(fit$hclust)
plot(yy)
bootstraps <- (round(fit$edges,2)*100)[,1:2]
yy<-as.phylo.hclust.with.nodenames(fit$hclust,
nodenames=bootstraps[,1])
#modified above to show au instead of bootstrap

sink(file="INPUT1-newick.newick")
# write newick file
write.tree(yy,tree.names=TRUE,digits=2)
sink()
plot(yy,show.node.label=TRUE,cex=0.5) #To show that the order is
correct
#saves plot with au values
savePlot(filename="INPUT1-plot",type=c("wmf"), device=dev.cur())
```
