# Supplementary material for: Total Ortholog Median Matrix as an alternative unsupervised approach for phylogenomics based on evolutionary distance between protein coding genes
Source: Sci Rep. 2021 Feb 15;11:3791. doi: 10.1038/s41598-021-81926-w (PMC7884790; doi:10.1038/s41598-021-81926-w)
Supplement: Supplementary file 3 — Supplementary Information 2. [file 41598_2021_81926_MOESM3_ESM.pdf]

Supplemental File 2 is deposited in DRYAD repository

(https://doi.org/10.5061/dryad.b1k526g) and available for Reviewers through the link https://datadryad.org/stash/share/_thib-jJ83k-pUKmabgh9HpjQNUoOWDis8kzGtRXXZI

Supplemental File 2: RSD resulting files (gene IDs) in compressed folders

The compressed folders named “RSD-Primates”, “RSD-Flies”, RSD-kinetoplastids”, contain text files with gene IDs resulted from RSD searches. Gene IDs for each paired species are tabulated in two columns, where first column indicates IDs from first species and second column indicates IDs from second species. Names of each paired species are abbreviated in the txt file name, using the first three letters for genus plus species, separated by hyphen, e.g.: the AOTNAN-CEBCAP-0.txt file presents gene IDs for *Aotus nancymaae* in the first column, while in the second column gene IDs belong to *Cebus capucinus*. The abbreviation for all organisms is supplied in Excel files Supplemental Tables S1, S2 and S3, under sheet “Abbreviation”.

File name: Supplemental_File2.tar.gz
