# Supplementary material for: Total Ortholog Median Matrix as an alternative unsupervised approach for phylogenomics based on evolutionary distance between protein coding genes
Source: Sci Rep. 2021 Feb 15;11:3791. doi: 10.1038/s41598-021-81926-w (PMC7884790; doi:10.1038/s41598-021-81926-w)
Supplement: Supplementary file 4 — Supplementary Information 3. [file 41598_2021_81926_MOESM4_ESM.pdf]

Total Ortholog Median Matrix as an alternative unsupervised approach for phylogenomics based on evolutionary distance between protein coding genes

Sandra Regina Maruyama, Luana Aparecida Rogerio, Patricia Domingues de Freitas, Marta Maria Geraldes Teixeira and José Marcos Chaves Ribeiro

Supplemental Material is deposited in DRYAD repository (https://doi.org/10.5061/dryad.b1k526g) and available for Reviewers through the link https://datadryad.org/stash/share/_thib-jJ83k-pUKmabgh9HpjQNUoOWDis8kzGtRXXZI

Supplemental Figures: S1, S2, S3, S4, S5, S6 and S7

S1-S6: Phylograms using different percentiles of the ranked ortholog pairs of 46 species of Kinetoplastida protozoa (S1, S2 and S4), different cutoff for E-value (S3) and different methods for orthology inference (S5 and S6).

S7: Bar graph showing the total number of orthologous identified by the RSD and OrthoMCL algorithms for 78 pairs of species combinations used in the analysis, based on 13 species with sequences retrieved from TriTryp database, as indicated in Table 1 ("Proteins sequence source" column). Intersections (shared orthologs) and unique orthologs were calculated with gene ID lists as input using Venn diagram tool (http://bioinformatics.psb.ugent.be/webtools/Venn/).

File name: Supplemental_Figures_S1_S2_S3_S4_S5_S6_S7.pdf

Supplemental Table S1: Kinetoplastida pairwise matrices

Excel spreadsheet containing resulting tables of pairwise orthologs data (pairwise matrices). Sheet "AA distance": amino acid distance obtained from median value calculated by RSD algorithm. Sheet "Number-50": total number of orthologs identified by RSD algorithm settings of 0.001 for the blast e-value of acceptance, and the value of 0.8 for the minimum ratio of the smallest sequence to the larger one.

File name: SupplementalTable_S1.xlsx

Supplemental Table S2: Hemataphagous Diptera pairwise matrices

Excel spreadsheet containing resulting tables of pairwise orthologs data (pairwise matrices). Sheet "AA distance": aminoacid distance obtained from median value calculated by RSD algorithm. Sheet "Number-50": total number of orthologs identified by RSD algorithm settings of of 0.001 for the blast e-value of acceptance, and the value of 0.8 for the minimum ratio of the smallest sequence to the larger one.

File name: SupplementalTable_S2.xlsx

Supplemental Table S3: Primates pairwise matrices

Excel spreadsheet containing resulting tables of pairwise orthologs data (pairwise matrices). Sheet "AA distance": aminoacid distance obtained from median value calculated by RSD algorithm. Sheet "Number-50": total number of orthologs identified by RSD algorithm settings of of 0.1 for the blast e-value of acceptance, and the value of 0.8 for the minimum ratio of the smallest sequence to the larger one.

File name: SupplementalTable_S3.xlsx

Supplemental Table S4: Comparison between orthology detection methods, RSD and OrthoMCL, using 13 species with protein sequences retrieved from TriTryp database.

Intersections (shared orthologs) and unique orthologs were calculated with gene ID lists from each method as input using Venn diagram tool (http://bioinformatics.psb.ugent.be/webtools/Venn/).

Supplemental File 1: R scripts

Scripts for hclust, pvclust and ape R packages used to build phylograms from amino acid distance matrices. File name: Supplemental_File1.pdf

Supplemental File 2: RSD resulting files (gene IDs) in compressed folders

The compressed folders named "RSD-Primates", "RSD-Flies", RSD-kinetoplastids", contain text files with gene IDs resulted from RSD searches. Gene IDs for each paired species are tabulated in two columns, where first column indicates IDs from first species and second column indicates IDs from second species. Names of each paired species are abbreviated in the txt file name, using the first three letters for genus plus species, separated by hyphen, e.g.: the AOTNAN-CEBCAP-0.txt file presents gene IDs for *Aotus nancymaae* in the first column, while in the second column gene IDs belong to *Cebus capucinus*. The abbreviation for all organisms is supplied in Excel files Supplemental Tables S1, S2 and S3, under sheet "Abbreviation".

File name: Supplemental_File2.tar.gz
